# Synthesis and Characterization of Transferrin Receptor-Targeted Peptide Combination SN-38 and Rucaparib Conjugate for the Treatment of Glioblastoma

**DOI:** 10.3390/pharmaceutics17060732

**Published:** 2025-06-02

**Authors:** Perpetue Bataille Backer, Simeon Kolawole Adesina

**Affiliations:** Department of Pharmaceutical Sciences, College of Pharmacy, Howard University, Washington, DC 20059, USA

**Keywords:** glioblastoma, peptide drug conjugate, SN-38, rucaparib, cathepsin B, combination therapy

## Abstract

**Background/Objectives:** Glioblastoma represents a particularly aggressive and fatal type of brain tumor. Peptide-drug conjugates, which offer the promise of traversing the blood-brain barrier to selectively accumulate in tumor tissues and precisely target cancer cells, are an active area of research. We present the synthesis and characterization of the T7 peptide (HAIYPRH) as a targeting ligand for the transferrin receptor, which is highly expressed on both the blood-brain barrier and glioma cells. **Methods**: Using the T7 peptide, the synthesis, characterization, and biological evaluation of a transferrin receptor-targeted, combination SN-38 and rucaparib peptide drug conjugate (T7-SN-38-rucaparib) are described. **Results**: The T7 peptide drug conjugate readily cleaved in the presence of exogenous cathepsin B, releasing the active drug payloads. In vitro experiments demonstrated potent cytotoxic effects of the T7 peptide drug conjugate on glioblastoma cells (IC_50_ = 22.27 nM), with reduced toxicity to non-cancerous HEK 293 cells (IC_50_ = 115.78 nM), indicating selective toxicity toward cancer cells. Further investigations revealed that blocking transferrin receptors with drug-free T7 peptide significantly reduced the conjugate’s cytotoxicity, an effect that could be reversed by introducing exogenous cathepsin B to the cells. **Conclusions**: These findings highlight the potential of glioblastoma-targeted delivery of SN-38 and rucaparib based on specific recognition of the transferrin receptor for transport across the blood-brain barrier, offering the prospect of reduced toxicity and selective killing of cancer cells. Additionally, since rucaparib does not cross the blood-brain barrier, this work is significant to facilitate the use of rucaparib for the treatment of brain tumors.

## 1. Introduction

Glioblastoma multiforme (GBM), a highly malignant brain tumor, presents a significant challenge in the field of oncology [1]. The median survival for patients with glioblastoma is around 14–16 months. Glioblastoma patients have a 5-year survival rate of about 5.8% and a recurrence rate of 90% [1,2]. The primary clinical treatment strategy is maximal surgical resection, radiotherapy, and concomitant temozolomide, with maintenance temozolomide (TMZ) chemotherapy. However, the efficacy of temozolomide treatment in achieving prolonged survival is hindered by the activation of various DNA repair pathways such as O-6-methylguanine-DNA methyltransferase (MGMT), MMR-mismatch repair, and BER-base excision repair pathways [3,4]. Several studies have provided substantial evidence supporting the central involvement of poly (ADP-ribose) polymerase (PARP) enzymes in coordinating the activation of the aforementioned DNA repair pathways, ultimately playing a role in the development of TMZ resistance [5,6,7,8].

In response to the complex nature of glioblastoma, there has been a notable shift in treatment strategies, recognizing the limitations of single-agent chemotherapy [9]. The imperative for combination therapies arises from the need to target multiple transcriptional networks and diverse cell populations, including cancer stem cells (CSCs), and to effectively combat tumor resurgence by resistant clonal populations [9,10]. One of the primary preclinically explored combination therapy strategies for GBM treatment involves pairing poly (ADP-ribose) polymerase inhibitors (PARPi) with TMZ [11,12,13].

PARP inhibitors (PARPi), such as rucaparib, inhibit PARP enzymatic activity, resulting in the accumulation of DNA double-strand breaks (DSBs) [14]. In cells lacking functional homologous recombination (HR), which is a DNA repair process essential for maintaining genomic stability and addressing complex DNA lesions, this accumulation of DSBs can lead to programmed cell death or apoptosis [14,15]. Since GBM is recognized for its absence of BRCA1/2 mutations (indicating proficiency in homologous recombination), the use of PARP inhibitors alone may not achieve the most effective anticancer results. However, when combined with other DNA-damaging agents, PARP inhibitors can selectively target and eliminate cancer cells in accordance with the principles of synthetic lethality, while preserving the integrity of normal cells with intact DNA repair mechanisms [3,16]. Thus, for gliomas, there could be potential to broaden the range of treatment options by incorporating PARPi alongside chemotherapy [3]. However, rucaparib’s lack of ability to traverse the blood-brain barrier (BBB), coupled with its vulnerability to transporters like P-glycoprotein and breast cancer resistance protein (BCRP), limits its clinical effectiveness in glioblastoma [16]. Thus, combining PARP inhibitors with DNA-damaging agents such as topoisomerase inhibitors offers a strategy to selectively eliminate cancer cells while preserving normal ones, in line with the principles of synthetic lethality.

7-Ethyl-10-hydroxycamptothecin (SN-38) is an active metabolite of irinotecan that demonstrates potent cytotoxicity against glioma cells [17]. However, its lipophilicity, high intravenous toxicity, and instability in physiological environments restrict its clinical application [17,18]. SN-38, as a topoisomerase I inhibitor, primarily impacts cells during the S and G2 phases of the cell cycle, leading to the formation of DNA single-strand breaks (SSBs) and the activation of DNA damage response proteins such as PARP [19,20]. PARPi, similar to rucaparib, hinders the repair of DNA SSBs and promotes their conversion to DSBs, thereby modulating DNA damage, increasing the fraction of tumor cells and/or time that tumor cells are arrested in the G2-M phase of the cell cycle, and augmenting the cells undergoing apoptosis [19,21]. While combination therapy proves more effective against solid tumors compared to monotherapy in the context of glioblastoma, the blood-brain barrier (BBB) remains a barrier to the efficacy of employing synergistic combinations of various therapeutic agents [10].

The blood-brain barrier controls the passage of solutes and chemicals between the peripheral circulation and central nervous system (CNS). This barrier presents a challenge, as it restricts access of chemotherapeutic agents to the brain and therefore impacts the effectiveness of chemotherapy, often necessitating high drug doses to achieve therapeutic levels in the treatment of brain tumors [22]. In high-grade gliomas such as glioblastoma, the integrity of the BBB is compromised, resulting in the formation of the blood-brain tumor barrier (BBTB) [23]. A distinctive feature of the BBB/BBTB is the presence of receptors, such as transferrin receptors (TfRs), crucial for iron transport into the brain tissue [24,25]. The specific abundance of TfRs on the endothelial cells of brain capillaries and overexpression on GBM cells compared to healthy cells render them an appealing target for precise drug delivery utilizing receptor-mediated transcytosis (RMT) [26].

Among various ligands for TfR targeting, transferrin (Tf) is notable for delivering drugs specifically to glioblastoma [27]. However, high levels of endogenous Tf can competitively inhibit Tf-modified delivery systems [27,28]. T7 peptide (His-Ala-Ile-Tyr-Pro-Arg-His; HAIYPRH) emerges as a promising alternative. T7 selectively targets TfR on the BBB, BBTB, and glioblastoma cells, exhibiting binding affinity comparable to Tf [29,30]. Its unique binding site on TfR, distinct from Tf, avoids competition with endogenous Tf and facilitates the uptake of T7-conjugates [27]. Numerous studies have explored conjugating T7 to different nanocarriers for targeted delivery to GBM [28,31,32,33]. Of these T7-targeted systems, targeted conjugates offer several advantages, such as controlled drug release, distinguishing them from other carrier systems that facilitate drug release by diffusion [34]. Peptide drug conjugates (PDCs) present a promising avenue, being small, non-immunogenic, and relatively stable. A PDC comprises a peptide targeting ligand for active targeting, a linker connecting the drug to the targeting ligand, and the toxin or drug payload [30,35,36]. Cleavable linkers, designed for controlled drug release within the tumor microenvironment, are crucial for minimizing impact on healthy tissues. Cathepsin B (Cat B), a lysosomal protease overexpressed in tumors, including glioblastoma, recognizes the valine-alanine (VA) dipeptide sequence, leading to linker cleavage and drug release in the tumor microenvironment [30,37].

We have previously reported the synthesis of T7 peptide-targeted delivery of SN-38 as monotherapy [30]. In the previous work, we confirmed that T7 binding to its receptor on U87MG glioma cells was essential for conjugate cytotoxicity in vitro. In the current work, the goal is to improve the T7 peptide-targeted delivery platform for the delivery of both SN-38 and rucaparib as a combination to increase cytotoxicity. This approach has the potential to enhance therapeutic efficacy in heterogenous cancers and reduce the challenges associated with chemoresistance. Additionally, T7 targeting offers the potential to transport rucaparib across the BBB as a consequence of binding to transferrin receptors that are expressed on the BBB. This is important, as rucaparib on its own does not cross the BBB and cannot be used for treatment that requires transport across the BBB. In our current work, we focus on synthesizing and characterizing a T7-SN-38-rucaparib targeted drug conjugate. Our investigation extends to assessing conjugate internalization and uptake by the TfR via blocking assays and evaluating cytotoxicity of the T7-targeted SN-38 and rucaparib conjugate in U87MG cells.

## 2. Experimental Section

### 2.1. Experimental Materials

Reagents were sourced from commercial suppliers and used as received. H-His(Trt)-2-Cl-Trt Resin and H-Val-2-Cl-Trt Resin were obtained from AAPPTEC (Louisville, KY, USA). Boc-L-alanine, Fmoc-L-alanine, Nim-Trityl-L-histidine tert-butyl ester hydrochloride, N^α^-Fmoc-N^ω^-(2,2,4,6,7-pentamethyldihydro-benzofuran-5-sulfonyl)-L-arginine Fmoc-L-isoleucine, Fmoc-protected O-tert-butyl-L-tyrosine, Fmoc-protected L-proline, Fmoc-protected S-trityl-L-cysteine, Fmoc-protected 6-aminohexanoic acid, 4-Aminobenzyl alcohol (PABA), N-Ethoxycarbonyl-2-ethoxy-1,2 dihydroquinoline (EEDQ), O-(7-benzotriazol-1-yl)-N,N,N’,N’-tetramethyluronium hexafluorophosphate (HATU), N,N’-Disuccinimidylcarbonate (DSC), 4-Dimethylaminopyridine (DMAP), Trifluoroacetic acid, N,N’-Diisopropylethylamine (DIPEA), and Piperidine were acquired from Chem Impex (Wood Dale, IL, USA). 7-Ethyl-10-hydroxycamptothecin (SN-38) was purchased from Biosynth (San Diego, CA, USA). N, N-Dimethylformamide, Dichloromethane (DCM), Methanol, Tetrahydrofuran, Acetonitrile, Ethyl Acetate, Hexane, Triisopropylsilane, Triethylamine (Et_3_N), Phosphorus tribromide (PBr_3_), Diethyl ether (Et_2_O), potassium carbonate, 18-crown-6, and silica gel were sourced from Sigma-Aldrich (Burlington, MA, USA). N_3_-PEG_4_-acid and DBCO-C_6_-acid were procured from Broad Pharm (San Diego, CA, USA). Maleimide-NH-PEG4-CH_2_CH_2_COOH and Fmoc-NH-PEG3-CH2COOH were obtained from PurePEG (San Diego, CA, USA). Rucaparib was purchased from BOC Biosciences (Shirley, NY, USA). The CyQUANT XTT Cell Viability Assay was obtained from Thermo fisher Scientific (Waltham, MA, USA).

### 2.2. Synthesis of N_3_-PEG_4_-Cys-PEG_3_-HAIYPRH (N_3_-Cys-T7 Peptide) (***1***)

**Figure 1:**
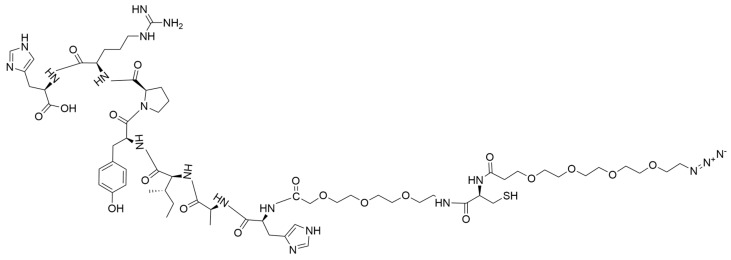
Structure of the T7-PEG_3_-Cys-PEG_4_-N_3_ targeting peptide (**1**).

N_3_-PEG_4_-Cys-PEG_3_-HAIYPRH (**1**) was synthesized by modifying a previously published method [30]. Our initial method had to be modified to introduce the amino acid cysteine (with a thiol functional group) to facilitate the incorporation of rucaparib as a second drug via a maleimide-coupled intermediate. Synthesis of N_3_-PEG_4_-Cys-PEG_3_-His-Ala-Ile-Tyr-Pro-Arg-His (**1**) was achieved by solid-phase peptide synthesis (SPPS). Initially, H-His (Trt)-2-Cl-Trt resin (5.0 g, 3.3 mmol) was allowed to swell in DMF for 2 h. Other amino acids were sequentially added as reported in our earlier publication. Each coupling was carried out in DMF using amino acid (2.5 eq, DIPEA (5 eq), and HATU (2.45 eq). Each coupling reaction was allowed to continue for 1 h. After each peptide coupling reaction, Fmoc protection was removed with 20% piperidine in DMF solution. The peptide chain was then extended by adding Fmoc-NH-PEG_3_-acid (1.35 eq), followed by Fmoc-L-Cys-(Trt)-OH. Subsequently, the N-terminus was capped with an azide-terminated polyethylene glycol linker (N_3_-PEG_4_-COOH) (Supporting Information Scheme S1). Post-reaction, the peptidyl resin was washed with DMF, DCM, and methanol successively and dried under vacuum overnight. N_3_-PEG_4_-Cys-PEG_3_-His-Ala-Ile-Tyr-Pro-Arg-His (Figure 1) was cleaved from the resin using a TFA/TIPS/H_2_O mixture (95:2.5:2.5) and the solvent was removed by rotary evaporation (Supporting Information Scheme S1). The resulting concentrate was precipitated in cold diethyl ether, yielding the product (**1**) as a yellow solid material (2.3 g, 48%). The N_3_-Cys-T7 peptide was characterized by analytical HPLC and ESI-MS. ESI-MS m/z 1459.1185; ([C_63_H_99_N_19_O_19_S+H]^+^ calculated 1459.6670) (Supporting Information, Figure S1). HPLC showed a single peak at a retention time (RT) of 4.65 min, confirming the compound’s purity (Supporting Information, Figure S2). Fourier transform infrared (FTIR) spectroscopy identified the functional groups present in compound **1** (Supporting Information, Figure S3). The compound was used without further purification.

### 2.3. Synthesis of DBCO-PEG_3_-Ahx-Val-Ala-PAB-SN-38 (***2***)

**Figure 2:**
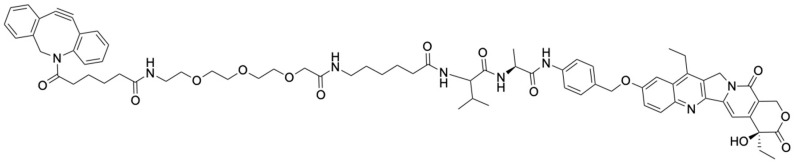
Structure of DBCO-PEG_3_-Ahx-Val-Ala-PAB-SN-38 (**2**).

DBCO-PEG_3_-Ahx-Val-Ala-PAB-SN-38 (**2**) (Figure 2) was synthesized in a multistep sequence following the previously reported method [30]. For detailed synthesis and characterization of these compounds, please refer to our published article on the single-drug conjugate [30].

### 2.4. Synthesis of Maleimide-PEG_4_-Ahx-Val-COOH (***3***)

**Figure 3:**
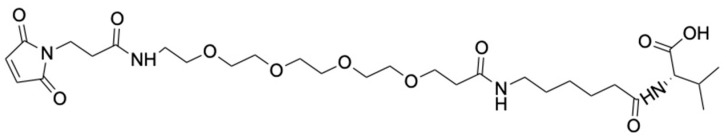
Structure of the Mal-PEG_4_-Ahx-Val-COOH (**3**).

Mal-PEG_4_-Ahx-Val-COOH (**3**) (Figure 3) was synthesized by modifying a published method [38]. H-Val-2-Cl-Trt Resin (5 g, 3.325 mmol/g) was allowed to swell in DMF for 2 h, followed by sequential couplings with Fmoc-6-aminohexanoic acid (2.5 eq) and Mal-PEG_4_-acid (1.5 eq) using DIPEA (5 eq) and HATU (2.45 eq) for 1 h. After the reactions, the peptidyl resin was washed in sequence using DMF, DCM, and methanol and dried under vacuum overnight. Mal-PEG_4_-Ahx-Val-COOH was cleaved from the resin using 1% TFA in DCM and concentrated *in vacuo*. Precipitation of the crude product in cold ether was performed, followed by drying under vacuum (Supporting Information Scheme S2). Maleimide-PEG_4_-Ahx-Val-COOH (Figure 3) was obtained as a golden viscous liquid (1.89 g, 90%) and characterized using ESI-MS and analytical HPLC. ESI-MS m/z 629.34; ([C_29_H_48_N_4_O_11_+H]^+^ calculated 629.73) (Supporting Information, Figure S4). HPLC showed a single peak at a retention time (RT) of 4.20 min, confirming the compound’s purity (Supporting Information, Figure S5).

### 2.5. Synthesis of Boc-Ala-PAB-Rucaparib (***4***)

**Scheme 1:**
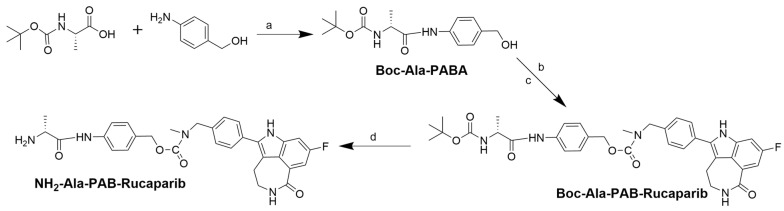
Synthesis of NH_2_-Ala-PAB-rucaparib. (a) EEDQ, DMF, 15 h. (b) DMAP, TEA, DSC, dry ACN, 3 h. (c) Rucaparib, dry DMF, 16 h. (d) 10% TFA in DCM, 1 h.

Boc-Ala-PAB-rucaparib (**4**) was synthesized by modifying a published method [39]. In brief, 4-dimethylaminopyridine (DMAP) (0.086 g, 0.71 mmol) and triethylamine (Et_3_N) (8.1 mmol, 1.14 mL) were added to Boc-Ala-PABA (0.8 g, 2.7 mmol) in anhydrous acetonitrile (ACN) (10 mL) under nitrogen. A solution of N, N′-disuccinimidyl carbonate (DSC) (0.905 g, 3.5 mmol) in dry ACN was added to the mixture with continuous stirring. Thin-layer chromatography (TLC) indicated the complete reaction of Boc-Ala-PABA with DSC after 3 h. Rucaparib (0.87 g, 2.7 mmol) in dry DMF was added to the reaction in an inert atmosphere and stirred for 16 h, after which the reaction was stopped, and the solvent was evaporated in vacuo. The obtained product was then purified using preparative HPLC (Scheme 1). Boc-Ala-PAB-rucaparib (**4**) (0.76 g, 44%) was characterized by ESI-MS and analytical HPLC. ESI-MS m/z 644.291; ([C_35_H_38_FN_5_O_6_+H]^+^ calculated 644.724) (Supporting Information, Figure S6). HPLC showed a single peak at a retention time (RT) of 7.16 min, confirming the compound’s purity (Supporting Information, Figure S7).

### 2.6. Synthesis of NH_2_-Ala-PAB-Rucaparib (***5***)

To synthesize NH_2_-Ala-PAB-rucaparib (**5**), Boc-Ala-PAB-rucaparib (0.8 g, 1.24 mmol) was dissolved in 10% TFA in DCM for Boc deprotection [40]. The solution was stirred for 1 h, and the reaction completion was monitored by ESI-MS. After completion of the deprotection reaction, the reaction solvent was removed *in vacuo*. NH_2_-Ala-PAB-rucaparib (**5**) was characterized by ESI-MS and analytical HPLC. ESI-MS m/z 544.247; ([C_30_H_30_FN_5_O_4_ + H]^+^ calculated 544.607) (Supporting Information, Figure S8). HPLC showed a single peak at a retention time (RT) of 5.30 min, indicating the compound’s purity (Supporting Information, Figure S9). It was used without further purification.

### 2.7. Synthesis of Mal-PEG_4_-Ahx-Val-Ala-PAB-Rucaparib (***6***)

**Figure 4:**
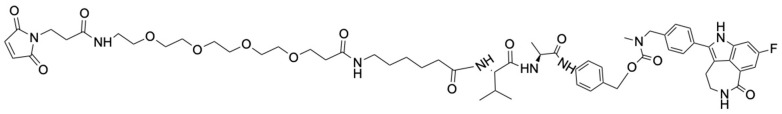
Structure of the Mal-PEG_4_-Ahx-Val-Ala-PAB-rucaparib (**6**).

To synthesize Mal-PEG_4_-Ahx-Val-Ala-PAB-rucaparib (**6**), Mal-PEG_4_-Ahx-Val-COOH (**3**) (0.65 g, 1.03 mmol) was dissolved in anhydrous DMF, and HATU (0.38 g, 1 mmol) and DIPEA (0.7 mL, 4.12 mmol) were added. After 15 min, NH_2_-Ala-PAB-rucaparib (**5**) (0.56 g, 1.03 mmol) was added, and reaction completion was monitored by HPLC. After 3 h, the solvent was removed by rotary evaporation, and the reaction mixture was purified by preparative HPLC to obtain Mal-PEG_4_-Ahx-Val-Ala-PAB-rucaparib (**6**) (Figure 4) as a yellow viscous liquid (0.47 g, 41%). This was characterized by ESI-MS and analytical HPLC. ESI-MS m/z 1154.91; ([C_59_H_76_FN_9_O_14_] calculated 1154.30) (Supporting Information, Figure S10). HPLC showed a single peak at RT of 6.03 min, confirming the compound’s purity (Supporting Information, Figure S11).

### 2.8. Synthesis of N_3_-PEG_4_-T7-Rucaparib (***7***)

**Figure 5:**
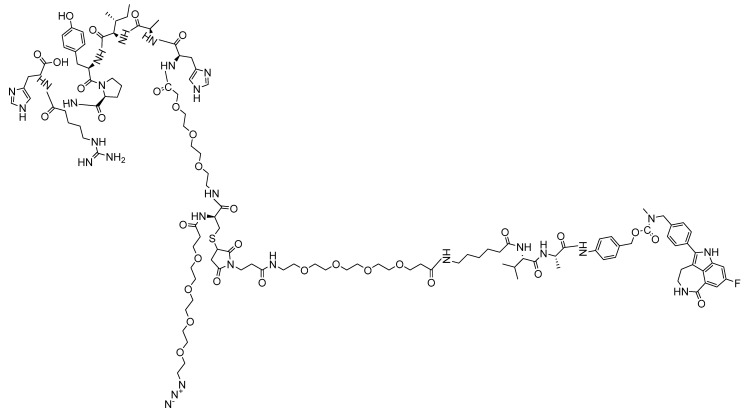
Structure of the N_3_-PEG_4_-T7-rucaparib (**7**).

To a solution of N_3_-PEG_4_-Cys-PEG_3_-His-Ala-Ile-Tyr-Pro-Arg-His (**1**) (0.3 g, 0.21 mmol) in DMF, Mal-PEG_4_-Ahx-Val-Ala-PAB-rucaparib (**6**) (0.24 g, 0.21 mmol) dissolved in dry DMF was added. A catalytic amount of DIPEA (1 wt%) was also added to the reaction mixture, which was stirred at room temperature under nitrogen [41]. The reaction was monitored by HPLC. After 4 h, the reaction was stopped. The reaction mixture was purified by preparative HPLC, evaporated to dryness, and N_3_-PEG_4_-T7-rucaparib (**7**) (Figure 5) was obtained as a yellow solid (0.17 g, 31%). ESI-MS of N_3_-PEG_4_-T7-rucaparib shows m/z 2614.5006; ([C_122_H_175_FN_28_O_33_S+H]^+^ calculated 2613.9714) (Supporting Information, Figure S12). Additionally, HPLC analysis of (**7**) shows a single peak with a retention time of 5.43 min, indicating that the pure compound was isolated (Supporting Information, Figure S13).

### 2.9. SPAAC of N_3_-PEG_4_-T7-Rucaparib (***7***) and DBCO-PEG_3_-Ahx-Val-Ala-PAB-SN-38 (***2***): Synthesis of Combination Drug Conjugate

**Figure 6:**
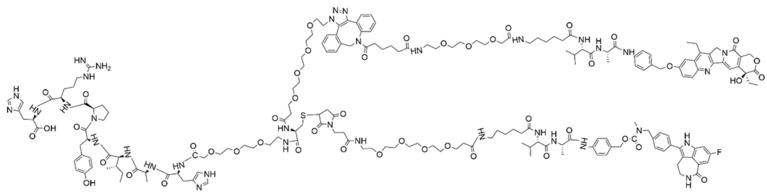
Structure of the T7-SN-38-rucaparib (**8**).

DBCO-PEG_3_-Ahx-Val-Ala-PAB-SN-38 (**2**) (0.02 g, 0.019 mmol) and N_3_-PEG_4_-T7-rucaparib (**7**) (0.05 g, 0.019 mmol) were dissolved in a total of 3 mL of methanol in a round-bottom flask. The SPAAC reaction was carried out by concentrating the solution using a rotary evaporator at 40 °C to facilitate the reaction. This process of redissolving the reactants in 3 mL of methanol followed by rotary evaporation was repeated 15 times to ensure the click reaction. The reaction mixture was purified by preparative HPLC, and the pure conjugate fraction was concentrated *in vacuo*. T7-SN-38-rucaparib (**8**) (Figure 6) was obtained as a yellow solid (0.026 g, 37%) and characterized using matrix-assisted laser desorption/ionization (MALDI) and analytical HPLC. MALDI results showed M/z 3902.98, M/2 1951.251, and M/3 1301.429 (Supporting Information, Figure S14). Additionally, HPLC showed a single peak at a retention time of 5.73 min, indicating that the pure compound was isolated (Supporting Information, Figure S15).

### 2.10. Evaluation of the In Vitro Cleavage Profile of T7-SN-38-Rucaparib

The cleavage profile of T7-SN-38-rucaparib was evaluated by following a published method [30]. Using this method, 11 μL of human liver cathepsin B stock (0.47 mg/mL, 324 U/mg, Merck Millipore) was activated in 27.2 μL of activation buffer (30 mM DTT, 15 mM EDTA in H_2_O) for 15 min. This mixture was added to 4.9 mL of reaction buffer (150 mM sodium acetate buffer, 12 mM EDTA, 24 mM DTT in H_2_O, pH 6.0). Cleavage was initiated by adding the cathepsin B solution to the T7-SN-38-rucaparib stock solution (**8**) in DMSO (final concentration 210 µM). The reaction was allowed to proceed at 37 °C, and samples were taken and analyzed by RP-HPLC at predetermined intervals (0–24 h). Changes in peak areas of the pure drugs and conjugate at their characteristic retention times to observe the disappearance of T7-SN-38-rucaparib and the appearance of free SN-38 and rucaparib over a 24 h period were monitored.

### 2.11. Cell Cultures

Human glioma cell line (U87MG) was obtained from American Type Culture Collection (ATCC). The cells were cultured in Eagle’s Minimum Essential Medium (EMEM) supplemented with 10% FBS (Corning, Manassas, VA, USA) and 1% penicillin-streptomycin (Sigma-Aldrich) at 37 °C at 5% CO_2_. Human embryonic kidney 293 cells (HEK 293), provided by Howard University’s Department of Biology, were maintained in Dulbecco’s Modified Eagle Medium (DMEM) with 10% FBS and 1% penicillin-streptomycin under the same conditions.

#### 2.11.1. T7-SN-38-Rucaparib Cytotoxicity Studies

U87MG cells (10,000 cells per well) were seeded in 96-well plates and allowed to attach for 24 h. The cells were then treated with either rucaparib + SN-38 solutions (drug ratio 1:1) or the T7-SN-38-rucaparib conjugate solutions at equivalent concentrations ranging from 5 nM to 160 nM and incubated at 37 °C. To evaluate cytotoxicity, XTT solution was added to each well per manufacturer protocols at 24 and 72 h after treatment. Following a 3-h incubation period, the absorbance of each well was measured at 450 nm using a Biotek ELx808 microplate reader (Lonza, Basel, Switzerland). Average absorbance values of the cells treated with different concentrations of test agents were normalized to those of the untreated cell controls. GraphPad Prism 10 was used to plot percent cell viability.

#### 2.11.2. Combination Index

CompuSyn^®^ software (Version 1.0) was used to evaluate and assess the interaction between SN-38 and rucaparib in U87MG cells. The combination index (CI) was calculated to determine the nature of the interaction: CI < 1 indicates synergy, CI = 1 indicates an additive effect, and CI > 1 indicates antagonism. For this study, constant concentration ratios between SN-38 and rucaparib (ratio 1:1) were maintained, and dose-effect curves were constructed using six data points. Viability was recorded as a percentage.

#### Rucaparib Cytotoxicity Studies

U87MG cells (10,000/well), seeded in 96-well plates, were allowed to attach for 24 h. The cells, incubated at 37 °C, were then treated with rucaparib solution at concentrations ranging from 5 nM to 160 nM. To evaluate cytotoxicity, XTT solution was added to each well at 24 and 72 h after treatment per manufacturer protocol. Following a 3-h incubation period, the absorbance of each well was measured at 450 nm using a Biotek ELx808 microplate reader (Lonza). The average absorbance of the cells treated with different concentrations of rucaparib were normalized to those of untreated cell controls. GraphPad Prism 10 was used to plot percent cell viability.

#### 2.11.3. T7-SN-38-Rucaparib Cytotoxicity Studies Using HEK-293 Cells

HEK-293 cells (15,000/well), seeded in 96-well plates, were allowed to attach for 24 h. Rucaparib + SN-38 solutions or the T7-SN-38-rucaparib conjugate solutions at equivalent concentrations (ranging from 5 nM to 160 nM) were added to the cells and incubated at 37 °C. To evaluate cytotoxicity, XTT solution was added to each well at 24 and 72 h after treatment per manufacturer protocol. Following a 3-h incubation period, the absorbance of each well was measured at 450 nm using a Biotek ELx808 microplate reader (Lonza). Average absorbance values of the cells treated with different concentrations of test agents were normalized to those of untreated cell controls, and percent cell viability was plotted using GraphPad Prism 10.

#### 2.11.4. T7 Competition Assay—Transferrin Receptor Blocking Studies

U87MG cells (1 × 10^4^), seeded in 96-well plates, were allowed to attach. After 24 h, the media was replaced with medium containing T7-PEG_4_-Cys-N_3_ (transferrin receptor targeting ligand) in a tenfold molar excess relative to the T7-SN-38-rucaparib conjugate for 1 h to block the transferrin receptor ligand binding site. Cells were subsequently treated with either rucaparib + SN-38 combination solutions or the T7-SN-38-rucaparib conjugate solutions at equivalent drug concentrations (range from 5 nM to 160 nM). To evaluate cytotoxicity, XTT solution was added to each well at 24 and 72 h after treatment per manufacturer protocol. Following a 3-h incubation period, the absorbance of each well was measured at 450 nm using a Biotek ELx808 microplate reader (Lonza). Average absorbance values of the cells treated with different concentrations of test agents were normalized to those of the untreated cell controls. GraphPad Prism 10 was used to plot percent cell viability.

#### 2.11.5. T7 Competition Assay—Exogenous Cathepsin B Studies

In these studies, blocking was done as reported for the transferrin receptor blocking studies above. Cells were subsequently treated with SN-38 solution (20 nM), SN-38 + rucaparib solution (20 nM each), T7-SN-38 solution (containing 20 nM of SN-38), and the T7-SN-38-rucaparib conjugate solution (containing 20 nM each of SN-38 and rucaparib). In an additional step, exogenous cathepsin B was added to the treated wells. In a parallel experiment, cells were treated with the same test agents at the same concentrations but without exogenous cathepsin B. XTT assays were conducted at 72 h following the manufacturer’s protocol for cytotoxicity. Percent cell viability was calculated, and the data were plotted using GraphPad Prism 10.

### 2.12. Statistical Analysis

IC_50_ values were calculated by concentration-response curve fitting using Microsoft Excel 16.77 (Microsoft Corp). Data represent mean ± standard deviation (SD) unless otherwise indicated. Differences between groups were determined by ANOVA, with a *p*-value < 0.05 deemed statistically significant.

## 3. Results

### 3.1. Design of T7 Peptide Combination Drug Conjugate

**Figure 7:**
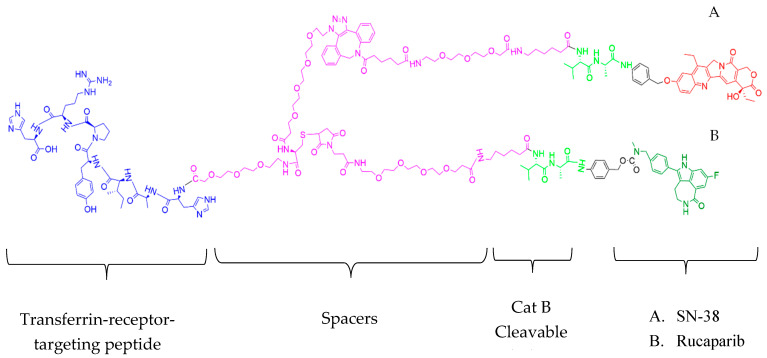
Structure of T7-SN-38-rucaparib. T7-SN-38-rucaparib contains a transferrin receptor targeting peptide, spacers, Cat B cleavable ValAla substrate, and self-immolative linker (PABA) linked to (**A**) SN-38 and (**B**) rucaparib.

**Figure 8:**
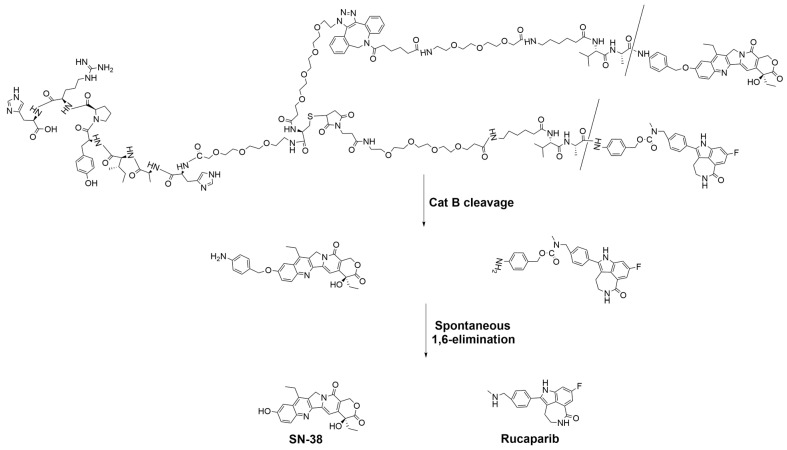
Proposed cleavage mechanism of T7-SN-38-rucaparib to release free SN-38 and free rucaparib.

The rational design of targeted conjugates is essential to fulfill peculiar, targeted drug delivery attributes. Figure 7 depicts the molecular structure of the T7-SN-38-rucaparib conjugate, which comprises several key elements: (i) a targeting peptide (HAIYPRH) for transferrin receptors; (ii) a spacer to prevent steric hindrance; (iii) a valine-alanine dipeptide protease recognizable sequence (sensitive to cleavage by cathepsin B); (iv) a self-immolative linker (PABA); and (v) combination anticancer agents (SN-38 and rucaparib). Generally, and by design, the conjugate binds to transferrin receptors that are highly expressed on the endothelial cells of brain capillaries (compared to much lower levels of expression in other tissues), facilitated by the T7 peptide’s affinity for TfR. This T7 binding is hypothesized to enable transport via transferrin receptor-mediated transcytosis across the BBB, facilitating conjugate entry into the brain parenchyma, where the conjugate selectively targets glioblastoma cells that are also known to overexpress TfR at significantly higher levels than healthy cells. Thus, upon binding to glioblastoma cells via the transferrin receptor, the conjugate is internalized, leading to cleavage by cathepsin B, followed by self-immolation via 1,6-elimination, activating SN-38 and rucaparib to induce their effects selectively to the cancer cells. Figure 8 illustrates the mechanism of SN-38 and rucaparib release from T7-SN-38-rucaparib.

### 3.2. Synthesis of the Dual Drug Conjugate (T7-SN-38-Rucaparib)

To synthesize the T7-SN-38-rucaparib conjugate, the peptide T7 was synthesized using solid-phase peptide synthesis (SPPS) (see Supporting Information, Scheme S1) and terminated with PEG_4_-azide. Confirmation of N_3_-PEG_4_-Cys-PEG_3_-HAIYPRH synthesis was achieved through mass spectrometry (m/z = 1459.1185) (Supporting Information, Figure S1) and FT-IR spectroscopy, which showed peaks around 2100 cm^−1,^ confirming the presence of the azide group, and at 2550 cm^−1,^ indicating the thiol (SH) group (Supporting Information, Figure S3). Thiol-maleimide chemistry facilitated the conjugation of maleimide-PEG_4_-Ahx-Val-Ala-rucaparib to N_3_-PEG4-Cys-PEG_3_-HAIYPRH, while SPAAC chemistry was employed to couple DBCO-PEG_3_-Ahx-Val-Ala-PAB-SN-38 to the azido group of the targeting peptide, resulting in the final T7-SN-38-rucaparib conjugate. Purification of the conjugate was carried out using preparative HPLC, and its characterization was confirmed by mass spectrometry (see Supporting Information, Figure S14) and RP-HPLC for purity (Supporting Information, Figure S15).

### 3.3. In Vitro Cleavage Studies of T7-SN-38-Rucaparib

**Figure 9:**
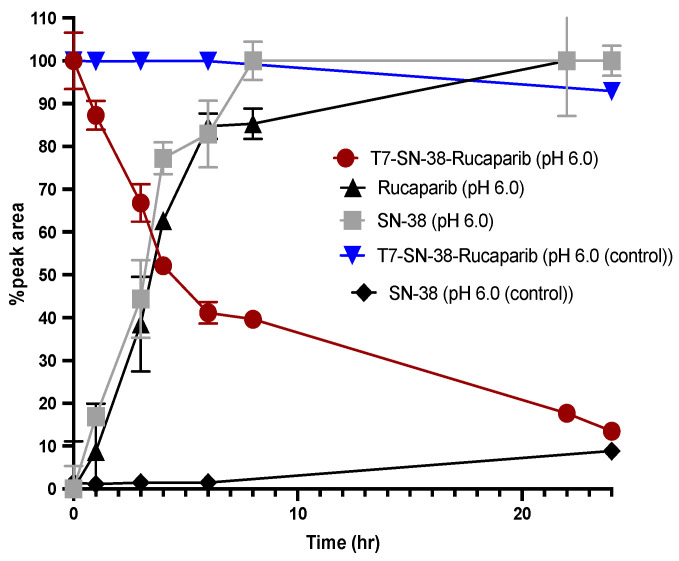
Plot of conjugate solution in the presence of cathepsin B (T7-SN-38-rucaparib; red line), appearance of parent drugs (SN-38; gray line) and (rucaparib; black line). Plot also showing control conjugate solution in the absence of exogenous cathepsin B (T7-SN-38-rucaparib; blue line) and appearance of free SN-38 in the absence of cathepsin B (SN-38; black line) (*n* = 3).

The activation and release of SN-38 and rucaparib from the T7-SN-38-rucaparib conjugate is critical for inducing cytotoxic effects in glioblastoma cells. Our hypothesis is that upon delivery to glioblastoma cells, T7-SN-38-rucaparib undergoes cleavage by cathepsin B, liberating free SN-38 and rucaparib (Figure 8). To assess the enzymatic release of the cytotoxic payload, exogenous cathepsin B was introduced to solutions of the conjugate, and aliquots were sampled at intervals over 24 h and analyzed by analytical RP-HPLC. The data reveals that at 24 h, about 87% of the T7-SN-38-rucaparib conjugate was cleaved by cathepsin B, releasing free SN-38 and rucaparib (Figure 9). Table 1 below shows the percentage of T7-SN-38-rucaparib cleaved at different sampling times.

**Figure 10:**
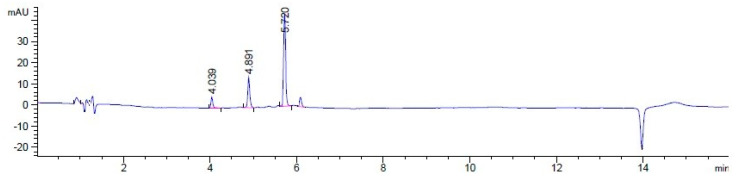
HPLC spectrum showing analysis of the one-hour sample in cleavage studies showing the peak of rucaparib (RT = 4.04 min), SN-38 (RT = 4.89 min), and the peak of unhydrolyzed conjugate (RT = 5.72 min).

**Figure 11:**
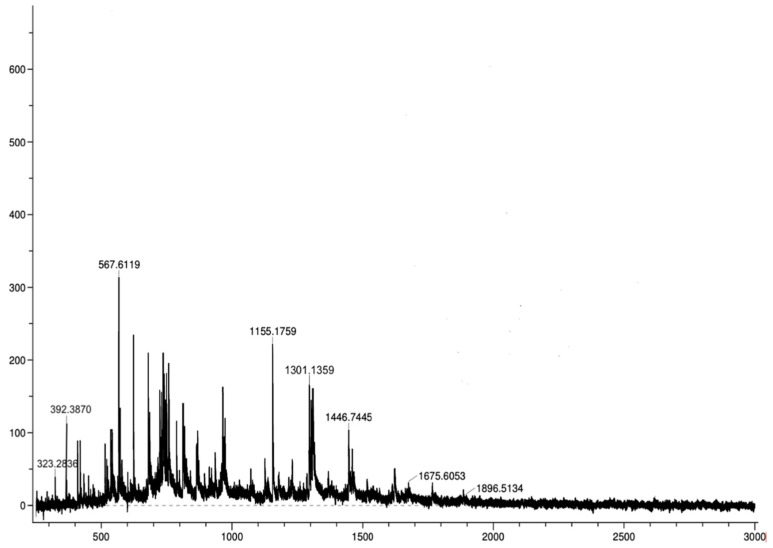
ESI-MS spectrum of a one-hour sample in cleavage studies of the T7-SN-38-rucaparib drug conjugate showing a mass of SN-38 m/z of 392.38 and rucaparib m/z of 323.28.

Additionally, HPLC analyses show that in the presence of cathepsin B, the dual-drug conjugate undergoes cleavage, yielding peaks corresponding to the retention times of free SN-38 (RT = 4.89 min), free rucaparib (RT = 4.04 min), and the parent conjugate itself (RT = 5.72 min) over 24 h (Figure 10). Over time, the conjugate peak area decreased while the peak areas of the free drugs increased. Mass spectrometry analysis confirmed the release of SN-38 (m/z = 392.38) and rucaparib (m/z = 323.28) from the T7-SN-38-rucaparib conjugate in the presence of cathepsin B (Figure 11), supporting our hypothesis. Furthermore, minimal release of free drugs (<10%) occurred when the conjugate was incubated in enzyme-free buffer at pH 6.0, indicating stability of the T7-SN-38-rucaparib conjugate.

### 3.4. Cell Culture Experiments

#### 3.4.1. Cytotoxicity Evaluation of Rucaparib on U87MG Cells

**Figure 12:**
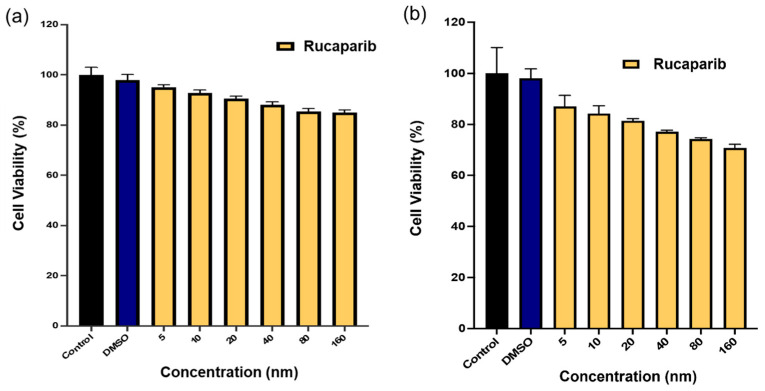
Percent cell viability data of rucaparib effect on U87MG cell lines after (**a**) 24 h exposure to treatment and (**b**) 72 h exposure to treatment. Media and DMSO solution in medium served as controls. Data represents mean ± std (*n* = 4).

The antiproliferative effect of rucaparib alone was examined using an XTT assay. In the range of concentrations examined (5 nM-160 nM), rucaparib did not display significant cytotoxic effects at 24 h (Figure 12a). However, over a 72-h period, rucaparib demonstrated slightly increased cytotoxicity on U87MG cells (Figure 12b).

#### 3.4.2. Cytotoxicity Evaluation of T7-SN-38-Rucaparib on U87MG Cells

**Figure 13:**
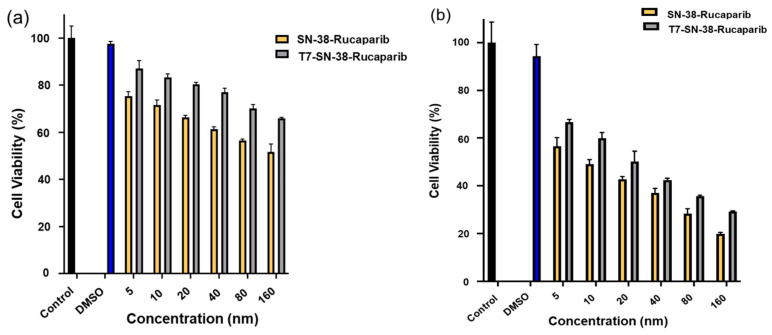
Percent cell viability data of SN-38-rucaparib and T7-SN-38-rucaparib effect on U87MG cell lines after (**a**) 24 h exposure to treatment and (**b**) 72 h exposure to treatment. DMSO solution in culture media and culture media served as controls. Data represents mean ± std (*n* = 4).

The XTT assay was employed to assess the cytotoxic effects of T7-SN-38-rucaparib, containing equivalent amounts of SN-38 and rucaparib as the SN-38 + rucaparib combination solution (SN-38-rucaparib), on U87MG cells across a range of concentrations (5 nM–160 nM). Results indicate that both formulations exhibited concentration- and time-dependent cytotoxicity against U87MG cells (Figure 13). At 24 h, the SN-38-rucaparib combination solution demonstrated significantly greater cytotoxicity compared to the conjugate (*p* < 0.05) (see Figure 13a). Over a 72 h period, both T7-SN-38-rucaparib and SN-38-rucaparib displayed considerable cytotoxic effects on U87MG cells (see Figure 13b). Additionally, the IC_50_ values for SN-38-rucaparib and T7-SN-38-rucaparib on U87-MG cells at 72 h were determined to be 9.73 nM and 22.27 nM, respectively.

#### 3.4.3. Assessment of Combination Index of SN-38-Rucaparib

The Chou-Talalay combination index (CI) method, developed by Chou in 2010, offers a quantifiable means to define synergism (CI < 1), an additive effect (CI = 1), or antagonism (CI > 1) in drug combinations [42]. According to the data in Table 2, all CI values were consistently less than 1, indicating a synergistic interaction.

#### 3.4.4. T7 Competition Assay: Transferrin Receptor Blocking Studies

**Figure 14:**
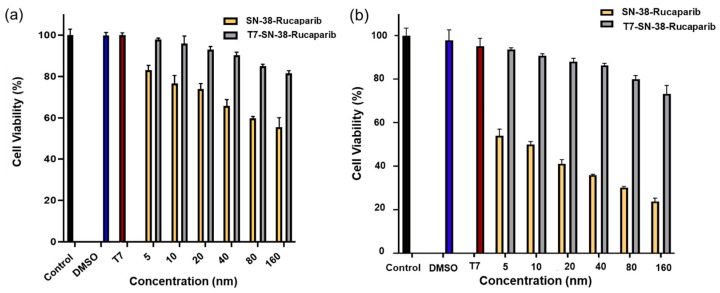
Percent cell viability data of SN-38-rucaparib and T7-SN-38-rucaparib effect on U87MG cell lines after (**a**) 24 h treatment of TfR-blocked cells and (**b**) 72 h treatment of TfR-blocked cells. DMSO solution in medium, culture media, and cells blocked with a tenfold molar excess of the T7 peptide served as controls. Data represents mean ± std (*n* = 4).

To assess the role of transferrin receptor-mediated endocytosis in the cytotoxicity of T7-SN-38-rucaparib, U87MG cells were pre-treated with excess N_3_-Cys-PEG_3_-T7 to block TfR receptors for 1 h. Subsequently, cells were exposed to equimolar concentrations of SN-38 + rucaparib (SN-38-rucaparib) or T7-SN-38-rucaparib conjugate solutions. Despite TfR blockade, concentration-dependent cytotoxicity was evident in cells treated with SN-38 + rucaparib solutions at 24 and 72 h (Figure 14). The calculated IC_50_ was 8.33 nM at 72 h (see Figure 14b). In addition, U87MG cells treated with the highest concentrations of T7-SN-38-rucaparib conjugate solution (160 nM) maintained approximately 80% cell viability, while only about 25% viability was observed in cells treated with SN-38-rucaparib control solutions at the same concentration for 72 h (see Figure 14b).

#### 3.4.5. Competition Assay: Validation of Importance of the Transferrin Receptor for Observed Cytotoxicity

To determine whether the cytotoxicity of the synthesized peptide drug conjugate relies on receptor-mediated endocytosis and intracellular cathepsin B cleavage for drug release or if it involves passive drug diffusion across cell membranes after extracellular cleavage, transferrin receptors on U87MG cells were saturated with excess targeting peptide, as described in the methods. Subsequently, U87MG cells with blocked transferrin receptors were treated with T7-SN-38-rucaparib (equivalent to 20 nM SN-38-rucaparib) and 20 nM SN-38-rucaparib alone as controls, both in the presence and absence of exogenous cathepsin B.

**Figure 15:**
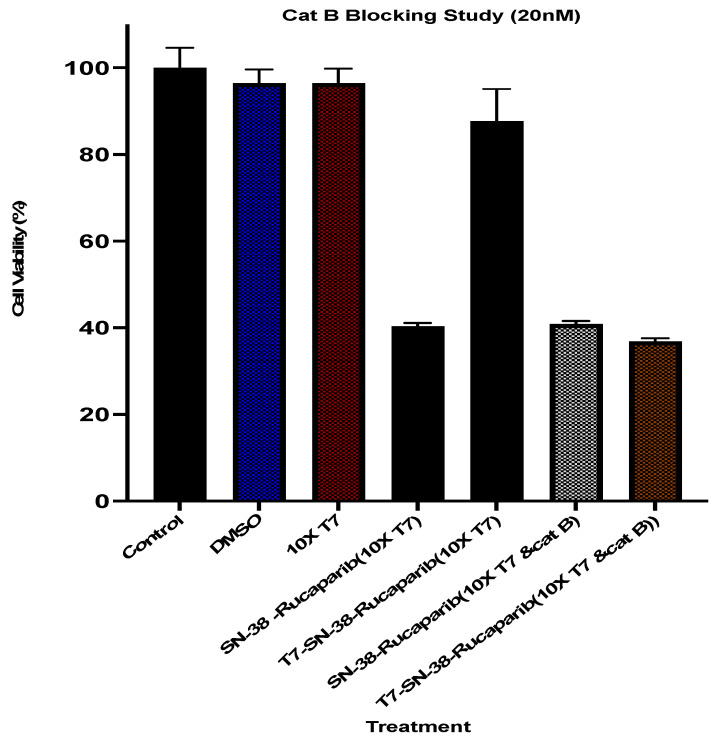
Percent cell viability data of U87MG cells preincubated with excess T7 targeting ligand (10×) and treated with culture media containing 20 nM SN-38-rucaparib solution and T7-SN-38-rucaparib containing 20 nM of the drugs in the presence and absence of exogenous cathepsin B after 72 h exposure to treatments. Controls are DMSO in culture media, culture media only, and a 10-fold molar excess of the T7 peptide in culture medium. Data represents mean ± std (*n* = 4).

As depicted in Figure 15, when U87MG cells with blocked transferrin receptors were exposed to a 20 nM solution of SN-38-rucaparib, the free drugs diffused into the cells, resulting in considerable cytotoxicity. Addition of exogenous cathepsin B to the combination drug solution did not alter cytotoxicity in U87MG cells, indicating cathepsin B-independent cytotoxicity. In contrast, cells with blocked transferrin receptors treated with T7-SN-38-rucaparib showed high percent cell viability (>85%) at 72 h (Figure 15), suggesting that cellular internalization of the conjugate and activation or intracellular drug release depends on binding to the TfR as a first step. To confirm this experimentally, cathepsin B was added to cells treated with T7-SN-38-rucaparib in blocking studies. The results show that the wells to which cathepsin B was exogenously added showed comparable cytotoxicity to the cells treated with free drugs (Figure 15). The exogenously administered cathepsin B facilitated extracellular conjugate cleavage and drug release, allowing drug entry into the cells via diffusion, similar to the drug solution. This confirms that the mechanism of site-specific drug delivery for the peptide drug conjugate relies on TfR targeting and the subsequent cathepsin B-mediated drug release (Figure 15).

#### 3.4.6. T7-SN-38-Rucaparib Cytotoxicity Against HEK-293 Cells

**Figure 16:**
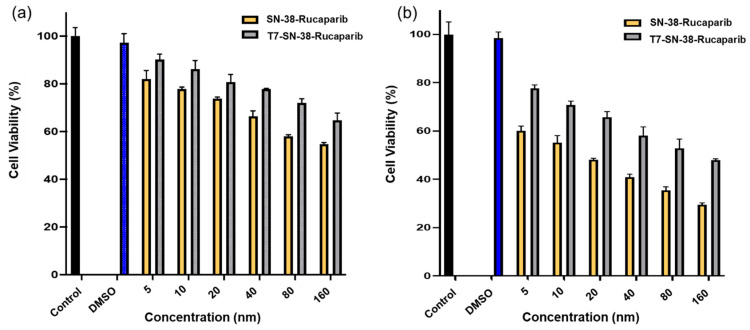
Percent viability data of SN-38-rucaparib and the T7-SN-38-rucaparib conjugate effect on HEK-293 cell lines after (**a**) 24 h exposure to treatment and (**b**) 72 h exposure to treatment. Controls represent culture media and DMSO solution in culture media. Data represent mean ± std (*n* = 4).

The cytotoxic effects of SN-38-rucaparib and T7-SN-38-rucaparib solutions, containing equivalent amounts of SN-38 and rucaparib as the mixture of free drugs, were evaluated in HEK-293 cells across a range of concentrations using the XTT assay. Both treatments exhibited time- and concentration-dependent cytotoxicity at 24 h and 72 h. At 24 h, T7-SN-38-rucaparib facilitated cell death (Figure 16a), with significantly lower cytotoxicity compared to free SN-38-rucaparib (*p* < 0.05). This was also the case at 72 h (Figure 16b). Furthermore, IC_50_ values for SN-38-rucaparib and T7-SN-38-rucaparib on HEK-293 cells at 72 h were determined. The IC_50_ value for SN-38-rucaparib was 16.11 nM, which was about 10-fold lower than the IC_50_ value of 115.78 nM determined for the T7-SN-38-rucaparib conjugate.

## 4. Discussion

Glioblastoma (GBM), an incurable and malignant form of cancer, poses significant challenges in treatment due to cell heterogeneity and chemotherapy resistance [43]. Overcoming these limitations often necessitates the use of multiple drugs or therapies [9]. Combination therapy, involving the simultaneous administration of drugs with different mechanisms of action (MOA), has emerged as a crucial strategy in cancer treatment to enhance efficacy [44]. Despite promising combinations such as PARP inhibitors with topoisomerase inhibitors, the inability of certain drugs, such as rucaparib, to cross the blood-brain barrier restricts their use in glioblastoma therapy. Recognizing the advantages of combination chemotherapy, a dual-drug targeted prodrug conjugate offers a potential solution, facilitating the co-delivery of drugs with distinct mechanisms and ensuring consistent biodistribution [44,45].

To address the blood-brain barrier challenge, our approach explores a novel dual drug conjugation strategy involving the coupling of SN-38 and rucaparib to the T7 peptide via valine-alanine, a Cat B recognition sequence. Leveraging the binding affinity of the T7 peptide to the transferrin receptor (TfR) overexpressed on the BBB and glioma cells, this approach aims to facilitate targeted drug payload delivery to glioblastoma, with subsequent internalization and Cat B-mediated cleavage ensuring site-specific drug release within cancer cells [46,47]. Modifying SN-38 and rucaparib with Cat B labile linkers ensures stability during circulation, allowing controlled release specifically within tumor cells and minimizing off-target effects [47,48].

In vitro cleavage studies using exogenous Cat B highlight the significance of the cathepsin B-sensitive linker in mediating the release of SN-38 and rucaparib from the conjugate. The observed enzymatic cleavage, coupled with the minimal cleavage in control experiments lacking cathepsin B, underscores the specificity and reliability of our approach.

The biological evaluation of the design attributes of the synthesized targeted conjugate focused on in vitro studies using U87MG, a malignant glioma cell line that inherently overexpresses the transferrin receptor (TfR) [49]. As a first step, the cytotoxic effect of rucaparib as a single agent was assessed on U87MG cells, revealing minimal toxicity to cells (Figure 12). This is consistent with its mechanism of action as a PARP inhibitor approved for targeted therapy and not as a directly acting cytotoxic agent to cells. In cancers where HR repair is impaired, such as those harboring mutations in BRCA1 and BRCA2 genes, cancer cells heavily rely on alternative DNA repair mechanisms, such as base excision repair (BER), to counteract DNA damage. Consequently, the inhibition of PARP activity by rucaparib induces synthetic lethality, as these cancer cells become highly vulnerable to DNA damage accumulation and subsequent cell death [3,15]. However, in glioma cells like U87MG, deficiencies in DNA repair pathways are not pronounced compared to other cancer types commonly associated with HR defects. Gliomas often exhibit a diverse genetic landscape, and although alterations in DNA repair genes can occur, they may not be as prevalent or functionally significant as in other cancer types [14]. Therefore, the observed minimal cytotoxicity of rucaparib in U87MG cells could be attributed to the relatively intact DNA repair mechanisms present in these cells, potentially conferring resistance to the effects of rucaparib.

In subsequent experiments, we explored the synergistic impact of combining rucaparib with SN-38 on cell viability. The results revealed that the combination of SN-38 and rucaparib exhibited considerably greater potency (Figure 13) compared to rucaparib alone (Figure 12). Furthermore, the concurrent administration of these drugs demonstrated synergistic effects, as revealed by combination index results, suggesting that the PARP inhibitor rucaparib, along with SN-38, disrupts the DNA strand rejoining process facilitated by topoisomerase I, thereby enhancing the accumulation of DNA damage and, consequently, amplifying the cytotoxic effects observed. This disruption augments the formation of DNA double-strand breaks, ultimately resulting in cell death [21]. This mechanism highlights the potential therapeutic benefits of combining these agents in glioma treatment strategies.

A concentration- and time-dependent decrease in U87MG cell viability was observed after treatment with T7-SN-38-rucaparib. The dual drug conjugate, T7-SN-38-rucaparib (IC_50_ = 22.27 nM), demonstrated greater potency in U87MG cells when compared to the single drug conjugate, T7-SN-38 (IC_50_ = 70.07 nM) [30]. Since rucaparib alone does not show appreciable toxicity to U87MG cancer cells (Figure 12), the combination with SN-38 enhances the efficacy of SN-38 in killing U87MG cells. This data, coupled with the combination index data, confirms the synergy of rucaparib and SN-38. Therefore, the combination therapy approach involving both SN-38 and rucaparib may hold promise for improving treatment outcomes in glioma.

Transferrin receptor blocking studies were conducted to evaluate the specificity of the drug conjugate for the transferrin receptor, establish a connection between receptor-mediated uptake and cytotoxic effectiveness, and discern any variances in the uptake mechanism between free SN-38-rucaparib and the T7-SN-38-rucaparib conjugate. Our results show that the cytotoxicity of the T7-targeted dual drug conjugate was significantly inhibited when the TfR receptors were blocked (Figure 15) compared to the cytotoxicity of the combination SN-38 + rucaparib solution, which was largely uninhibited. This difference in cytotoxic effects can be attributed to the mechanism of cellular uptake, where internalization of the T7-SN-38-rucaparib conjugate was hindered by excess free T7 targeting ligand, disrupting receptor-mediated endocytosis and subsequent drug release by intracellular cathepsin B cleavage. Additional experiments were conducted to evaluate the specificity and significance of cathepsin B cleavage to cytotoxicity, confirm the involvement of transferrin receptor-ligand interactions in uptake and subsequent therapeutic effectiveness, and confirm the pivotal role of the transferrin receptor in targeted therapy. Despite the blockade of the TfR as a result of preincubation with the free targeting ligand, U87MG cells exhibited considerable cytotoxicity when treated with T7-SN-38-rucaparib in the presence of exogenous cathepsin B, similar to the cytotoxic effect observed with free drug (Figure 15). This observation is because cleavage and release of free drugs occurred in the cell growth media, followed by diffusion into the cells, similar to the mechanism of free drugs in solution. These results underscore the critical design features of the targeted delivery system, highlighting the importance of intracellular localization for cathepsin B cleavage and efficient drug release.

To correlate TfR overexpression to cellular uptake and to determine the effect of the conjugate on non-cancerous cells, conjugate cytotoxicity was evaluated in human embryonic kidney 293 (HEK-293) cells to assess the drug conjugate cytotoxicity on “normal cells”. HEK-293 cells are widely recognized as a standard model for normal human cells in laboratory studies due to their low levels of transferrin receptors [50]. Cytotoxicity studies were carried out using this cell line to determine the effectiveness of the conjugate in targeting cancer cells while preserving the integrity of healthy cells. The objective is to evaluate the effectiveness of the conjugated drugs against malignant tissue while minimizing any adverse effects on normal cellular structures.

Cytotoxicity data show that both T7-SN-38-rucaparib and SN-38-rucaparib displayed time-dependent and concentration-dependent cytotoxicity in HEK-293 cells (Figure 16). Furthermore, the IC_50_ values obtained for SN-38-rucaparib and T7-SN-38-rucaparib in HEK-293 cells at 72 h were determined. The obtained IC_50_ value for SN-38-rucaparib was 16.11 nM, which is about 10 times lower than the IC_50_ value of 115.78 nM obtained for the T7-SN-38-rucaparib conjugate. Additionally, an IC_50_ of 22.27 nM was obtained for U87MG cells treated with the T7-SN-38-rucaparib conjugate. Put together, these findings show that the drug conjugate displays cytotoxicity towards normal cells at elevated concentrations compared to non-cancerous cells. This observation highlights a promising aspect of the potential of the T7-SN-38-rucaparib conjugate, generally referred to as “differential cytotoxicity” or “selective cytotoxicity”. This characteristic of the conjugate suggests that the conjugate possesses the ability to discriminate between cancerous and healthy cells. The difference in toxicity between cancerous and non-cancerous cells could be as a result of limited intracellular uptake in non-cancerous cells as a consequence of limited expression of TfR. This selectivity could potentially allow the conjugate to exert its cytotoxic effects more prominently on cancer cells while sparing normal cells, particularly at lower concentrations [51]. This attribute of the targeted conjugate holds significant implications for cancer treatment strategies. It implies the existence of a therapeutic window, a range of concentrations where the drug can effectively target and eliminate cancer cells while minimizing adverse effects on healthy tissues. This selective action could enhance the efficacy of the treatment while reducing the risk of harmful side effects, improving patient outcomes, and potentially widening the scope for therapeutic interventions in various types of cancer. Thus, the identification of this “differential cytotoxicity” underscores the promising prospects of the drug conjugate as a targeted and potentially safer approach for glioma therapy.

Generally, challenges to the use of PDCs for cancer treatment have been identified. PDCs are reported to have a short half-life in circulation as a consequence of degradation by proteases. Additionally, they are rapidly cleared by the kidneys as a result of their low molecular weight [52,53]. However, it is important to note that the low molecular weight of PDCs is advantageous as it facilitates improved penetration of tumors compared to other delivery systems such as antibody drug conjugates and vesicular delivery systems [54]. Thus, the potential disadvantages of peptide conjugate delivery systems must be weighed against their drug delivery advantages. For this work, such delivery advantages include ensuring similar biodistribution of rucaparib and SN-38, facilitating their uptake across the BBB as a result of TfR targeting, and allowing for synergy in the treatment of glioblastoma. Data obtained show the potential of glioma-targeted delivery of SN-38 and rucaparib for transport across the blood-brain barrier, offering the prospect of reduced toxicity and enhanced selective killing of cancer cells.

With the promising results obtained in this report, which show lower IC_50_ values with the combination therapy compared to monotherapy, further evaluations will seek to provide evidence of transendothelial transport and investigate delivery across the BBB, followed by pharmacokinetic and efficacy studies in animal models of glioblastoma.

## 5. Conclusions

By connecting small molecule drugs to targeting peptides through bio-cleavable linkers, peptide drug conjugates keep the drug inactive until they reach the intended site of action. Once binding to the target receptor takes place, cellular internalization occurs where the conjugate undergoes proteolytic processing to release the active drugs. In this study, a peptide targeting the transferrin receptor (T7 sequence: HAIYPRH) was employed as the carrier to leverage the high expression of transferrin on the blood-brain barrier (BBB) and its overexpression on glioma cells for site-specific drug delivery. In this work, T7-SN-38-rucaparib was constructed with the T7 peptide linked to the cytotoxic payload through a cathepsin B cleavable peptide linker, serving as the mechanism of drug release. In vitro cleavage studies (pH 6.0) confirmed cathepsin B-specific cleavage and release of SN-38 and rucaparib. The potency (IC_50_ = 22.27 nM) of the conjugate was evaluated in vitro to determine cytotoxicity in U87MG cancer cell lines. Blocking of TfR on U87MG cells using free targeting ligand shows reduced cytotoxicity of the conjugate on U87MG cells, indicating the essential role of transferrin receptors in cellular uptake. Moreover, introducing exogenous cathepsin B to cells with previously blocked transferrin receptors reversed the “blocking” effect, confirming that the conjugate releases active drug only in the presence of cathepsin B.

Furthermore, the cytotoxicity of the conjugate was assessed on non-cancerous (HEK-293) cells, yielding an IC_50_ value of 115.78 nM, indicating relative toxicity to non-cancerous cells at higher concentrations compared to cancerous U87MG cells (IC_50_ = 22.27 nM). This is advantageous and suggests that the peptide drug conjugate can mitigate off-target effects and cause minimal harm to healthy tissues at lower concentrations that are toxic to cancer cells. This differential toxicity indicates that the conjugate may be selective for cancer cells when administered at lower concentrations, facilitating effective tumor treatment while sparing normal cells. These findings underscore the translational potential of utilizing T7 as a targeting ligand in developing peptide-drug conjugates, providing a site-specific therapeutic approach for treatment of brain cancer.

## Figures and Tables

**Table 1:** Percent of T7-SN-38-rucaparib cleaved at different sampling times.

Time (h)	Percent Cleaved
0	0.0
1	17.8
4	43.8
8	61.6
24	87.3
26	96.0

**Table 2:** Calculated CI values for SN-38-rucaparib.

	Total Dose (nM)	CI Value
1	5	0.19701
2	10	0.18736
3	20	0.17750
4	40	0.20417
5	80	0.13751
6	160	0.07244

## Data Availability

The authors confirm that the data supporting the findings of this study are available within the article and its Supplementary Materials. Data are also available on request from the authors.

## Supplementary Materials

The following supporting information can be downloaded at: https://www.mdpi.com/article/10.3390/pharmaceutics17060732/s1, Scheme S1: Synthesis of T7-PEG_3_-Cys- PEG_4_-N_3_ (**1**); Scheme S2: Synthesis of Maleimide-PEG_4_-Ahx-Val (**7**); Figure S1: ESI-MS of N_3_-Cys-PEG_3_-HAIYPRH (**1**); Figure S2: RP-HPLC analysis of N_3_-Cys-PEG_3_-HAIYPRH (**1**); Figure S3: FTIR of Azide-Cys-PEG_3_-HAIYPRH (**1**); Figure S4: ESI-MS of Mal-PEG_4_-Ahx-Val (**3**); Figure S5: RP-HPLC of Mal-PEG_4_-Ahx-Val (**3**); Figure S6: ESI-MS of Boc-Ala-PAB-Rucaparib (**4**); Figure S7: RP-HPLC analysis of Boc-Ala-PAB-Rucaparib (**4**); Figure S8: ESI-MS of NH_2_-Ala-PAB-Rucaparib (**5**); Figure S9: RP-HPLC analysis of NH_2_-Ala-PAB-Rucaparib (**5**); Figure S10: ESI-MS of Mal-PEG_4_-Ahx-Val-Ala-PAB-Rucaparib (**6**); Figure S11: RP-HPLC analysis Mal-PEG_4_-Ahx-Val-Ala-PAB-Rucaparib (**6**); Figure S12: ESI-MS of N_3_-PEG_4_-T7-Rucaparib (**7**); Figure S13: RP-HPLC analysis of N_3_-PEG_4_-T7-Rucaparib (**7**); Figure S14: MALDI-TOF mass spectrum of T7-SN-38-Rucaparib conjugate (**8**); Figure S15: RP-HPLC analysis of T7-SN-38-Rucaparib (**8**).

## References

[1] Yeini E., Ofek P., Albeck N., Rodriguez Ajamil D., Neufeld L., Eldar-Boock A., Kleiner R., Vaskovich D., Koshrovski-Michael S., Dangoor S.I. (2021). Targeting glioblastoma: Advances in drug delivery and novel therapeutic approaches. Adv. Ther..

[2] Pineda E., Domenech M., Hernández A., Comas S., Balaña C. (2023). Recurrent glioblastoma: Ongoing clinical challenges and future prospects. OncoTargets Ther..

[3] Sim H.W., Galanis E., Khasraw M. (2022). PARP inhibitors in glioma: A review of therapeutic opportunities. Cancers.

[4] Liu D., Dai X., Tao Z., Zhou H., Hong W., Qian H., Cheng H., Wang X. (2023). Advances in blood–brain barrier-crossing nanomedicine for anti-glioma. Cancer Nanotechnol..

[5] Trivedi R.N., Almeida K.H., Fornsaglio J.L., Schamus S., Sobol R.W. (2005). The role of base excision repair in the sensitivity and resistance to temozolomide-mediated cell death. Cancer Res..

[6] Zhang X., Zhang W.E.I., Cao W.D., Cheng G., Zhang Y.Q. (2012). Glioblastoma multiforme: Molecular characterization and current treatment strategy. Exp. Ther. Med..

[7] Ohba S., Yamashiro K., Hirose Y. (2021). Inhibition of DNA repair in combination with temozolomide or dianhydrogalactiol overcomes temozolomide-resistant glioma cells. Cancers.

[8] Fahrer J., Christmann M. (2023). DNA alkylation damage by nitrosamines and relevant DNA repair pathways. Int. J. Mol. Sci..

[9] Ghosh D., Peng X., Leal J., Mohanty R.P. (2018). Peptides as drug delivery vehicles across biological barriers. J. Pharm. Investig..

[10] Zhao M., van Straten D., Broekman M.L.D., Préat V., Schiffelers R.M. (2020). Nanocarrier-based drug combination therapy for glioblastoma. Theranostics.

[11] Majd N.K., Yap T.A., Koul D., Balasubramaniyan V., Li X., Khan S., Gandy K.S., Yung W.A., De Groot J.F. (2021). The promise of DNA damage response inhibitors for the treatment of glioblastoma. Neuro-Oncol. Adv..

[12] Wu S., Li X., Gao F., De Groot J.F., Koul D., Yung W.A. (2021). PARP-mediated PARylation of MGMT is critical to promote repair of temozolomide-induced O6-methylguanine DNA damage in glioblastoma. Neuro-Oncology.

[13] Hwang K., Lee J.H., Kim S.H., Go K.O., Ji S.Y., Han J.H., Kim C.Y. (2021). The combination PARP inhibitor olaparib with temozolomide in an experimental glioblastoma model. In Vivo.

[14] Bisht P., Kumar V.U., Pandey R., Velayutham R., Kumar N. (2022). Role of PARP inhibitors in glioblastoma and perceiving challenges as well as strategies for successful clinical development. Front. Pharmacol..

[15] Chan W.Y., Brown L.J., Reid L., Joshua A.M. (2021). PARP inhibitors in melanoma—An expanding therapeutic option?. Cancers.

[16] Zhang S., Peng X., Li X., Liu H., Zhao B., Elkabets M., Liu Y., Wang W., Wang R., Zhong Y. (2021). BKM120 sensitizes glioblastoma to the PARP inhibitor rucaparib by suppressing homologous recombination repair. Cell Death Dis..

[17] Khaiwa N., Maarouf N.R., Darwish M.H., Alhamad D.W., Sebastian A., Hamad M., Omar H.A., Orive G., Al-Tel T.H. (2021). Camptothecin’s journey from discovery to WHO Essential Medicine: Fifty years of promise. Eur. J. Med. Chem..

[18] Dai Y., Qian M., Li Y. (2023). Structural Modification Endows Small-Molecular SN38 Derivatives with Multifaceted Functions. Molecules.

[19] Silpa N., Qiu-Xu T., Jagadish K., Jingquan W., Yehuda G.A., Charles R.A.J., Zhe-Sheng C. (2019). Poly (ADP-ribose) polymerase (PARP) inhibitors as chemosensitizing compounds for the treatment of drug-resistant cancers. J. Mol. Clin. Med..

[20] Han S., Lim K.S., Blackburn B.J., Yun J., Putnam C.W., Bull D.A., Won Y.W. (2022). The potential of topoisomerase inhibitor-based antibody–drug conjugates. Pharmaceutics.

[21] Tentori L., Graziani G. (2005). Chemopotentiation by PARP Inhibitors in Cancer Therapy. Pharmacol. Res..

[22] Jena L., McErlean E., McCarthy H. (2020). Delivery across the blood-brain barrier: Nanomedicine for glioblastoma multiforme. Drug Deliv. Transl. Res..

[23] Belykh E., Shaffer K.V., Lin C., Byvaltsev V.A., Preul M.C., Chen L. (2020). Blood-brain barrier, blood-brain tumor barrier, and fluorescence-guided neurosurgical oncology: Delivering optical labels to brain tumors. Front. Oncol..

[24] Pulgar V.M. (2019). Transcytosis to cross the blood brain barrier, new advancements and challenges. Front. Neurosci..

[25] Parrasia S., Szabò I., Zoratti M., Biasutto L. (2022). Peptides as pharmacological carriers to the brain: Promises, shortcomings and challenges. Mol. Pharm..

[26] Ayloo S., Gu C. (2019). Transcytosis at the blood–brain barrier. Curr. Opin. Neurobiol..

[27] Ramalho M.J., Loureiro J.A., Coelho M.A., Pereira M.C. (2022). Transferrin receptor-targeted nanocarriers: Overcoming barriers to treat glioblastoma. Pharmaceutics.

[28] Kuang Y., An S., Guo Y., Huang S., Shao K., Liu Y., Li J., Ma H., Jiang C. (2013). T7 peptide-functionalized nanoparticles utilizing RNA interference for glioma dual targeting. Int. J. Pharm..

[29] Papademetriou I.T., Porter T. (2015). Promising approaches to circumvent the blood–brain barrier: Progress, pitfalls and clinical prospects in brain cancer. Ther. Deliv..

[30] Bataille Backer P., Adekiya T.A., Kim Y., Reid T.E.R., Thomas M., Adesina S.K. (2024). Development of a Targeted SN-38-Conjugate for the Treatment of Glioblastoma. ACS Omega.

[31] Zong T., Mei L., Gao H., Shi K., Chen J., Wang Y., Zhang Q., Yang Y., He Q. (2014). Enhanced glioma targeting and penetration by dual-targeting liposome co-modified with T7 and TAT. J. Pharm. Sci..

[32] Bi Y., Liu L., Lu Y., Sun T., Shen C., Chen X., Chen Q., An S., He X., Ruan C. (2016). T7 peptide-functionalized PEG-PLGA micelles loaded with carmustine for targeting therapy of glioma. ACS Appl. Mater. Interfaces.

[33] Cheng X., Yu D., Cheng G., Yung B.C., Liu Y., Li H., Kang C., Fang X., Tian S., Zhou X. (2018). T7 peptide-conjugated lipid nanoparticles for dual modulation of Bcl-2 and Akt-1 in lung and cervical carcinomas. Mol. Pharm..

[34] Wang Y., Cheetham A.G., Angacian G., Su H., Xie L., Cui H. (2017). Peptide–drug conjugates as effective prodrug strategies for targeted delivery. Adv. Drug Deliv. Rev..

[35] Hoppenz P., Els-Heindl S., Beck-Sickinger A.G. (2020). Peptide-drug conjugates and their targets in advanced cancer therapies. Front. Chem..

[36] Chavda V.P., Solanki H.K., Davidson M., Apostolopoulos V., Bojarska J. (2022). Peptide-drug conjugates: A new hope for cancer management. Molecules.

[37] Bargh J.D., Isidro-Llobet A., Parker J.S., Spring D.R. (2019). Cleavable linkers in antibody–drug conjugates. Chem. Soc. Rev..

[38] Coin I., Beyermann M., Bienert M. (2007). Solid-phase peptide synthesis: From standard procedures to the synthesis of difficult sequences. Nat. Protoc..

[39] Akinboye E.S., Rosen M.D., Bakare O., Denmeade S.R. (2017). Anticancer activities of emetine prodrugs that are proteolytically activated by the prostate specific antigen (PSA) and evaluation of in vivo toxicity of emetine derivatives. Bioorganic Med. Chem..

[40] Liu L., Xie F., Xiao D., Xu X., Su Z., Wang Y., Fan S., Zhou X., Li S. (2021). Synthesis and evaluation of highly releasable and structurally stable antibody-SN-38-conjugates. Drug Deliv..

[41] Ziaei E., Saghaeidehkordi A., Dill C., Maslennikov I., Chen S., Kaur K. (2019). Targeting triple negative breast cancer cells with novel cytotoxic peptide–doxorubicin conjugates. Bioconjugate Chem..

[42] Chou T.C. (2010). Drug combination studies and their synergy quantification using the Chou-Talalay method. Cancer Res..

[43] Davis M.E. (2016). Glioblastoma: Overview of disease and treatment. Clin. J. Oncol. Nurs..

[44] Nilchan N., Li X., Pedzisa L., Nanna A.R., Roush W.R., Rader C. (2019). Dual-mechanistic antibody-drug conjugate via site-specific selenocysteine/cysteine conjugation. Antib. Ther..

[45] Duarte D., Vale N. (2022). Evaluation of synergism in drug combinations and reference models for future orientations in oncology. Curr. Res. Pharmacol. Drug Discov..

[46] Rao J.S. (2003). Molecular mechanisms of glioma invasiveness: The role of proteases. Nat. Rev. Cancer.

[47] Whang C.H., Yoo E., Hur S.K., Kim K.S., Kim D., Jo S. (2018). A highly GSH-sensitive SN-38 prodrug with an “OFF-to-ON” fluorescence switch as a bifunctional anticancer agent. Chem. Commun..

[48] Goldenberg D.M., Sharkey R.M. (2019). Antibody-drug conjugates targeting TROP-2 and incorporating SN-38: A case study of anti-TROP-2 sacituzumab govitecan. mAbs.

[49] Koneru T., McCord E., Pawar S., Tatiparti K., Sau S., Iyer A.K. (2021). Transferrin: Biology and use in receptor-targeted nanotherapy of gliomas. ACS Omega.

[50] Wang J., Tian S., Petros R.A., Napier M.E., DeSimone J.M. (2010). The complex role of multivalency in nanoparticles targeting the transferrin receptor for cancer therapies. J. Am. Chem. Soc..

[51] Li J., Gu Y., Zhang W., Bao C.Y., Li C.R., Zhang J.Y., Liu T., Li S., Huang J.X., Xie Z.G. (2019). Molecular mechanism for selective cytotoxicity towards cancer cells of diselenide-containing paclitaxel nanoparticles. Int. J. Biol. Sci..

[52] Fu C., Yu L., Miao Y., Liu X., Yu Z., Wei M. (2023). Peptide–drug conjugates (PDCs): A novel trend of research and development on targeted therapy, hype or hope?. Acta Pharm. Sin. B.

[53] Wang M., Liu J., Xia M., Yin L., Zhang L., Liu X., Cheng Y. (2024). Peptide-drug conjugates: A new paradigm for targeted cancer therapy. Eur. J. Med. Chem..

[54] Li S., Zhao H., Mao X., Fan Y., Liang X., Wang R., Xiao L., Wang J., Liu Q., Zhao G. (2019). Transferrin Receptor Targeted Cellular Delivery of Doxorubicin via a Reduction-Responsive Peptide-Drug Conjugate. Pharm. Res..

