# Supplementary material for: Synthesis and Characterization of Transferrin Receptor-Targeted Peptide Combination SN-38 and Rucaparib Conjugate for the Treatment of Glioblastoma
Source: Pharmaceutics. 2025 Jun 2;17(6):732. doi: 10.3390/pharmaceutics17060732 (PMC12196469; doi:10.3390/pharmaceutics17060732)
Supplement: Supplementary file 1 [file pharmaceutics-17-00732-s001.zip › pharmaceutics-3618766-supplementary.pdf]

pharmaceutics

MDPI

Supplementary Materials: Synthesis and Characterization of Transferrin Receptor-Targeted Peptide Combination SN-38 and Rucaparib Conjugate for the Treatment of Glioblastoma

Perpetue Bataille Backer and Simeon Kolawole Adesina

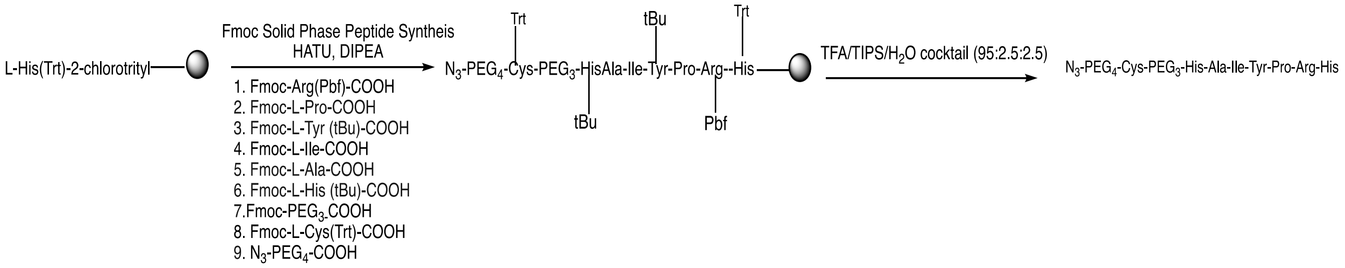

Scheme 1. Synthesis of T7-PEG$_3$-Cys-PEG$_4$-N$_3$ (**1**).

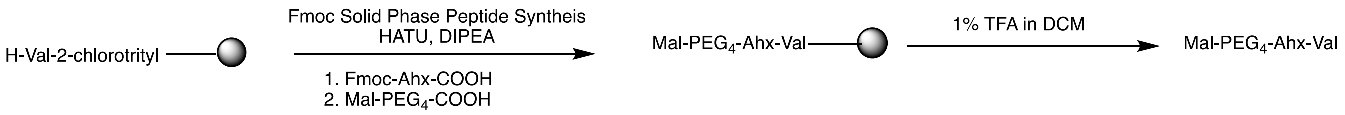

Scheme 2. Synthesis of Maleimide-PEG$_4$-Ahx-Val (**7**).

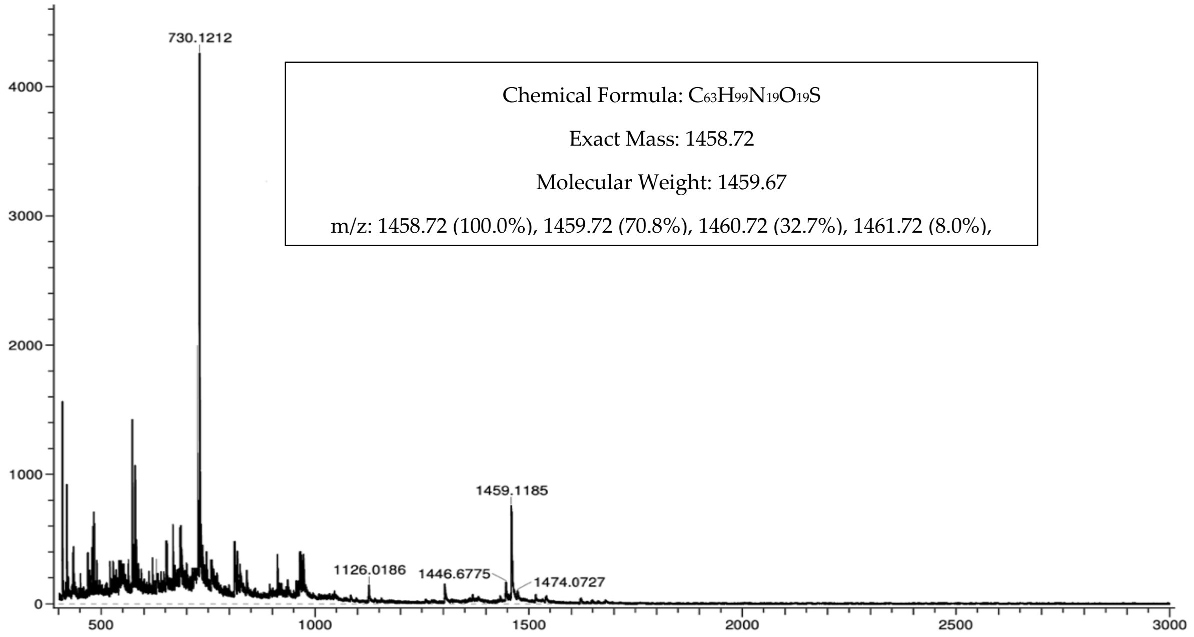

Figure S1. ESI-MS of N$_3$-Cys-PEG$_3$-HAIYPRH (**1**) m/z 1459.11; calcd. (M+H)1459.67.

 https://doi.org/10.3390/xxxxx

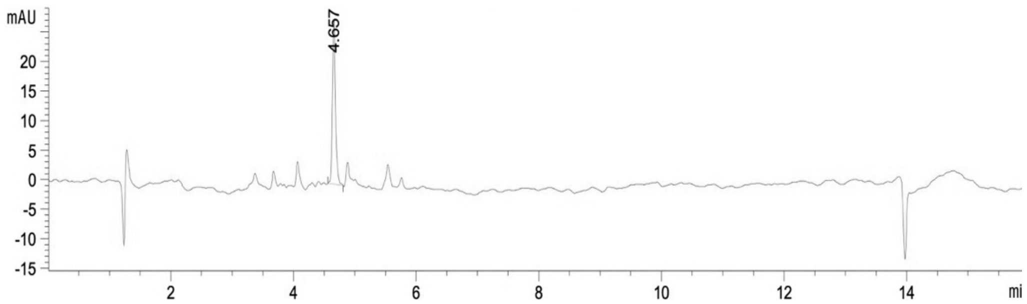

Figure S2. RP-HPLC analysis of N_3-Cys-PEG$_3$-HAIYPRH (**1**) showing isolation of compound 1 with retention time of 4.65 minutes.

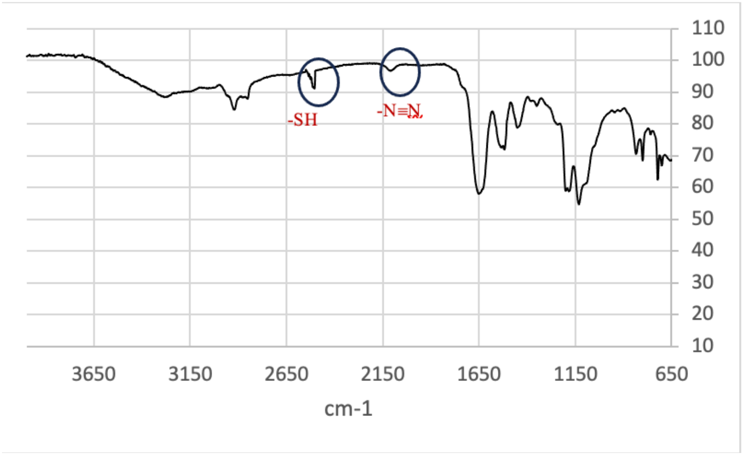

Figure S3. FTIR of Azide-Cys-PEG$_3$-HAIYPRH (**1**) confirms the termination of the T7 peptide with an azido group with a peak around 2100cm^{-1} and thiol group at around 2550 cm^{-1}.

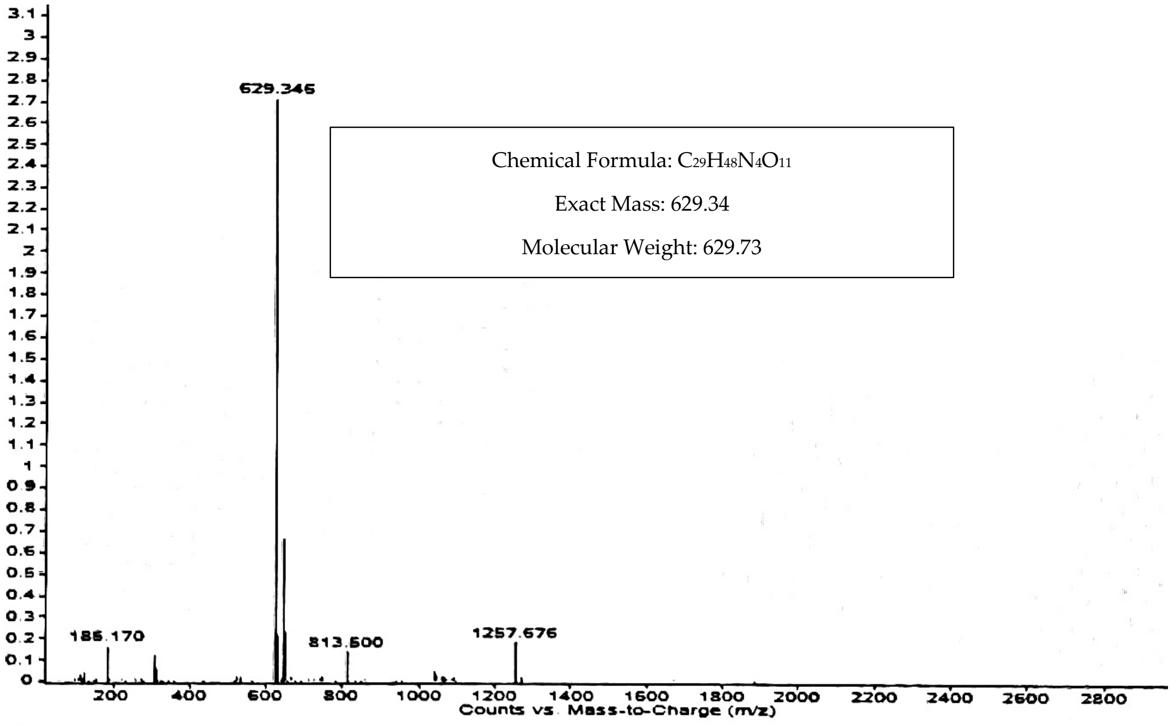

Figure S4. ESI-MS of Mal-PEG$_4$-Ahx-Val (**7**), m/z 629.34; calcd. (M+H) 629.73.

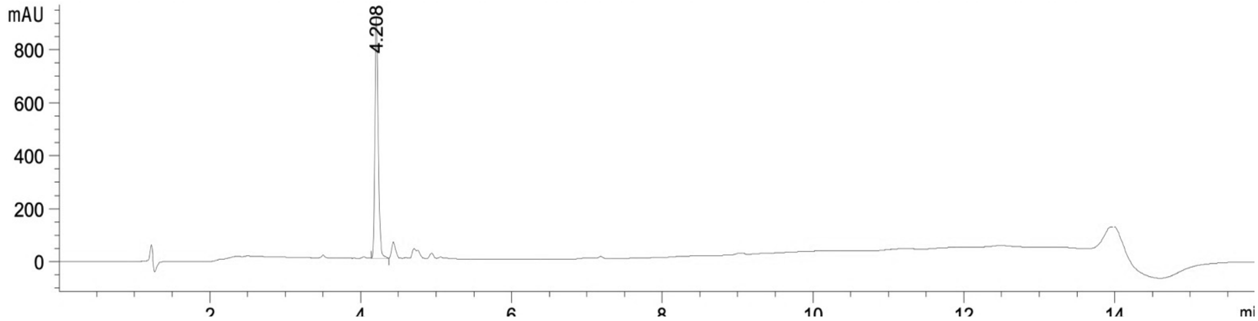

Figure S5. RP-HPLC of Mal-PEG$_4$-Ahx-Val (**7**) showing isolation of a pure compound with retention time of 4.20 minutes.

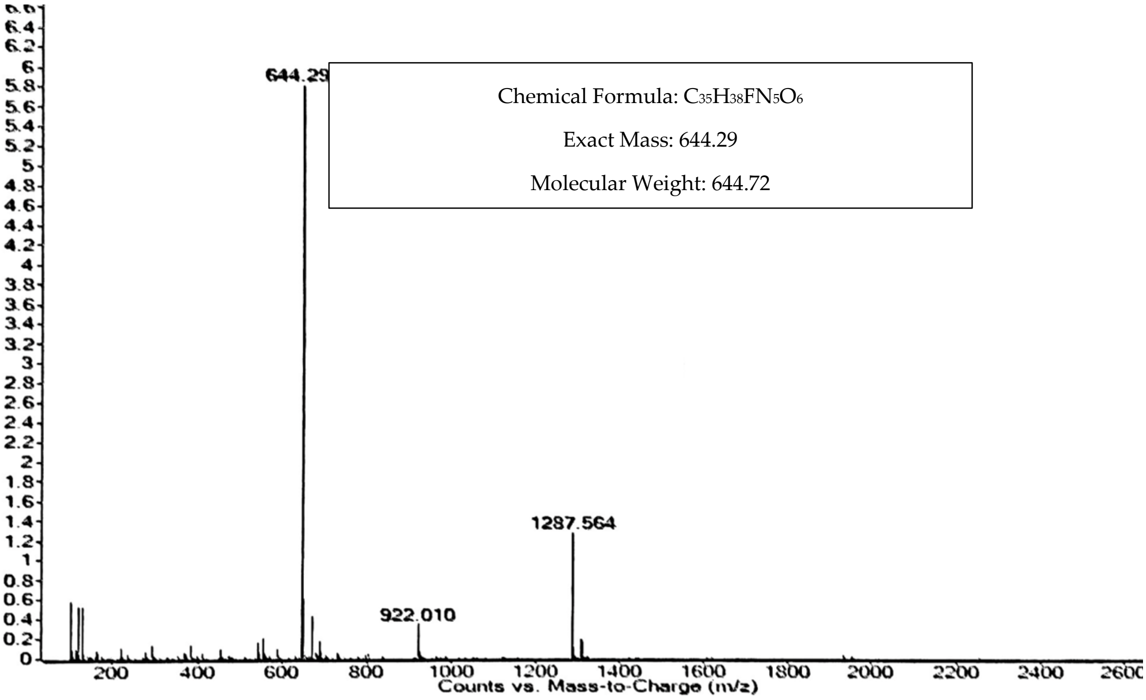

Figure S6. ESI-MS of Boc-Ala-PAB-Rucaparib (**8**), m/z 644.29; calcd.(M+H) 644.72.

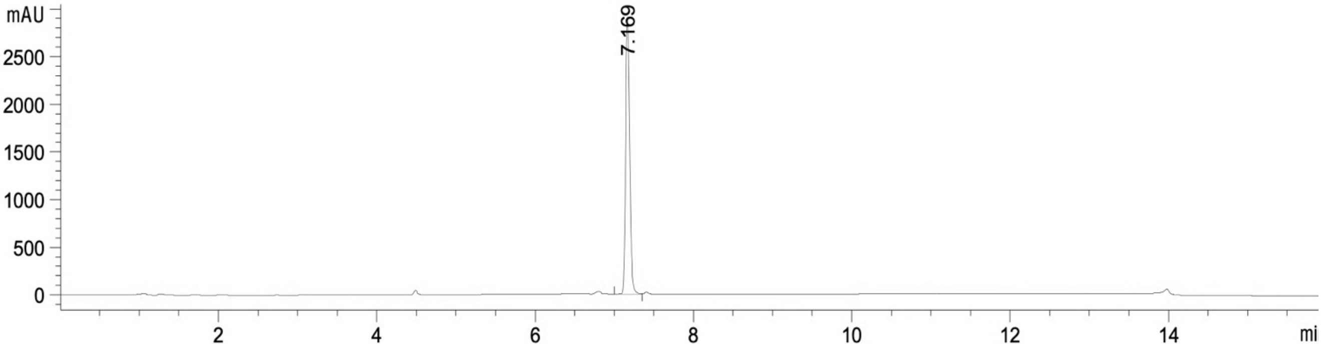

Figure S7. RP-HPLC analysis of Boc-Ala-PAB-Rucaparib (**8**), showing isolation of a pure compound with retention time of 7.16 minutes.

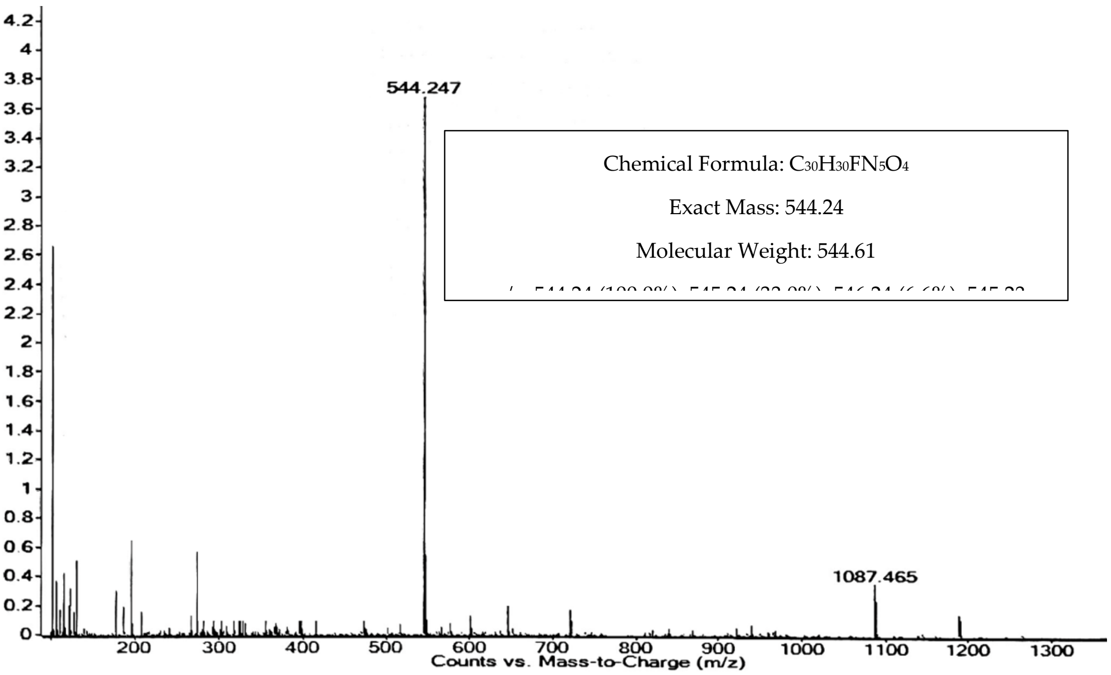

Figure S8. ESI-MS of NH_2-Ala-PAB-Rucaparib (**9**) m/z 544.24;calcd. (M+H) 544.61.

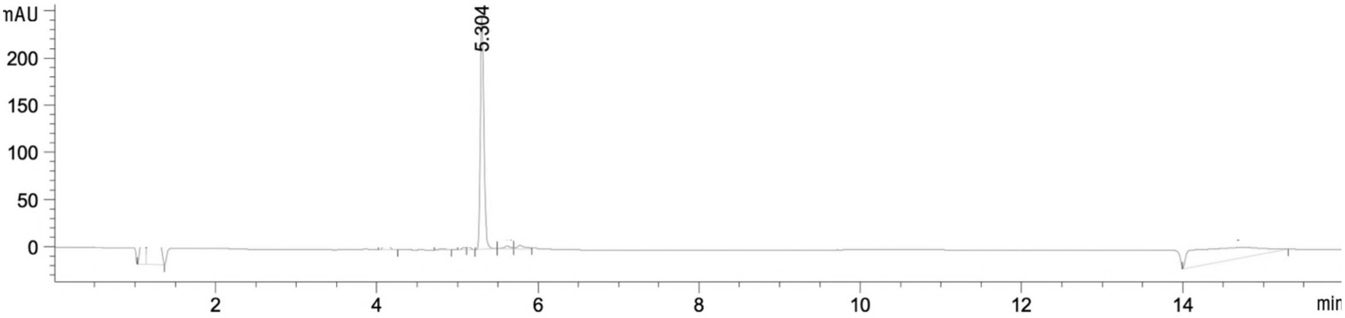

Figure S9. RP-HPLC analysis of NH_2-Ala-PAB-Rucaparib (**9**), showing isolation of a pure compound with retention time of 5.30 minutes.

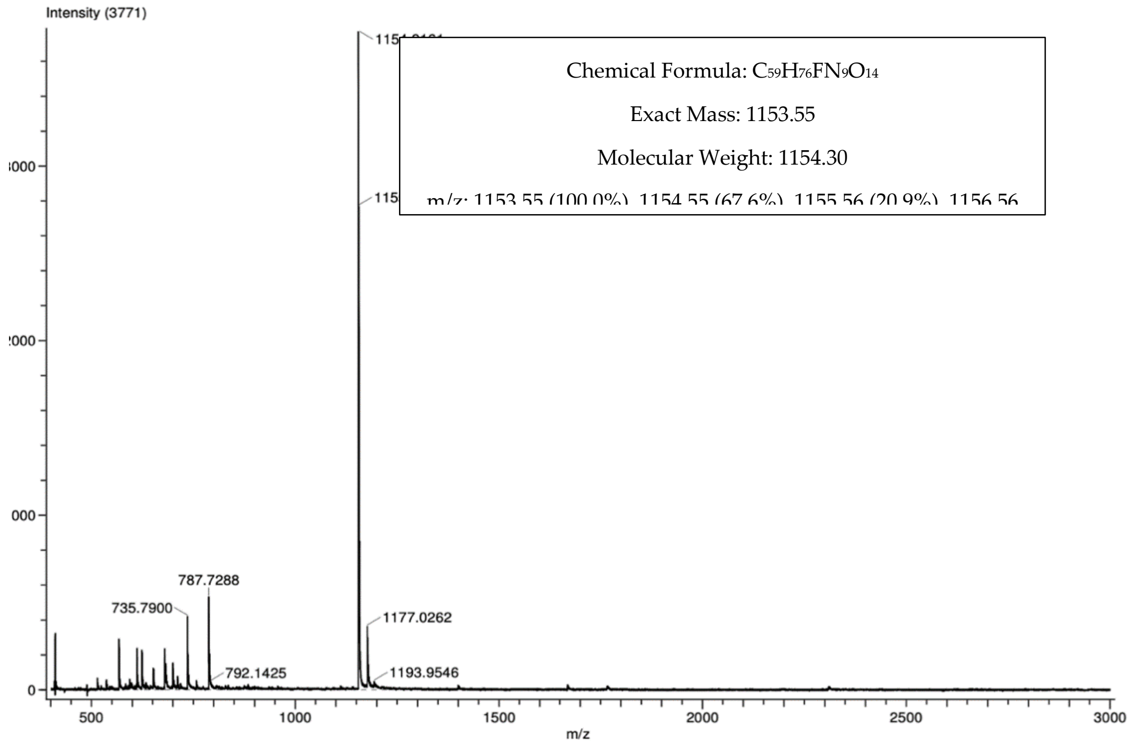

Figure S10. ESI-MS of Mal-PEG$_4$-Ahx-Val-Ala-PAB-Rucaparib (**10**) m/z1154.91;calcd.(M+H)1154.30.

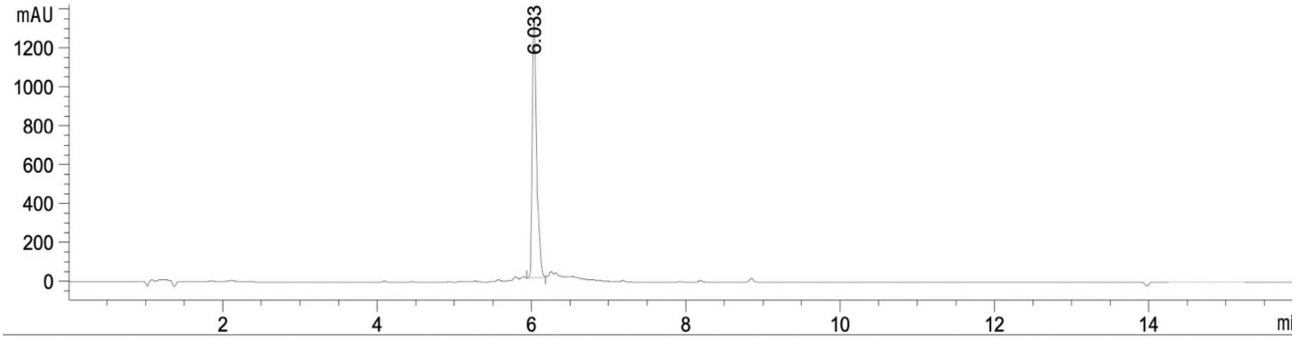

Figure S11. RP-HPLC analysis Mal-PEG$_4$-Ahx-Val-Ala-PAB-Rucaparib (**10**) showing isolation of a pure compound with retention time 6.03 minutes.

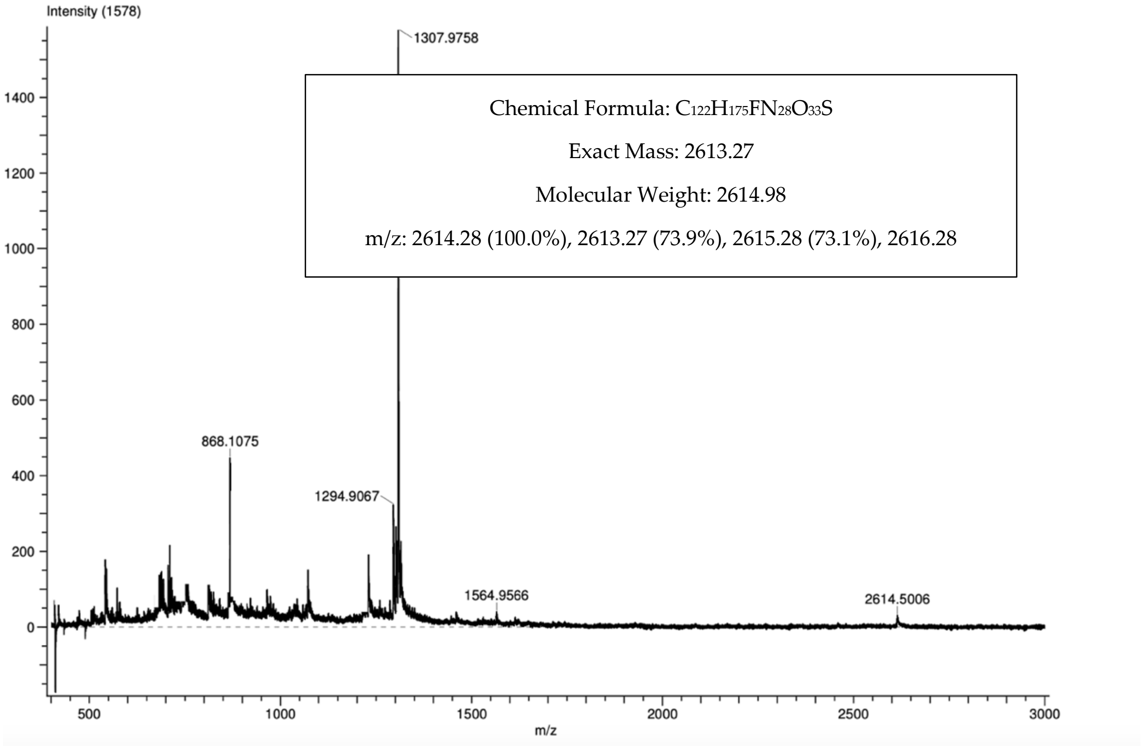

Figure S12. ESI-MS of N_3-PEG_3T7-Rucaparib (**11**) m/z 2614.50; mass/2=1307.97; calcd.2614.98.

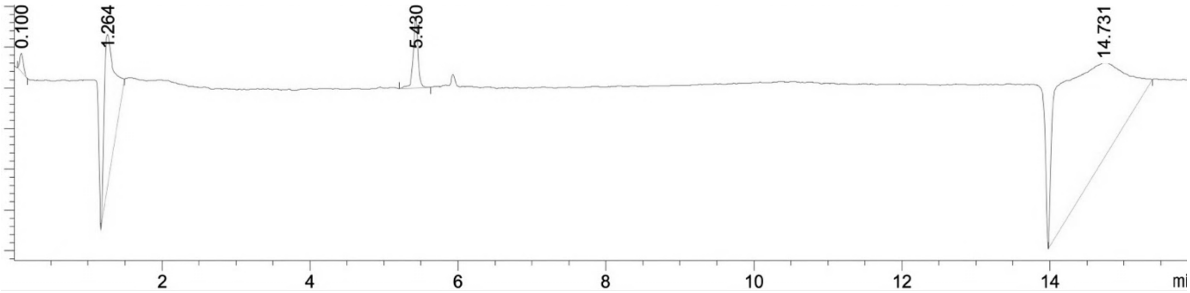

Figure S13. RP-HPLC analysis of N_3-PEG_3T7-Rucaparib (**11**) showing isolation of a pure compound with retention time 5.43 min.

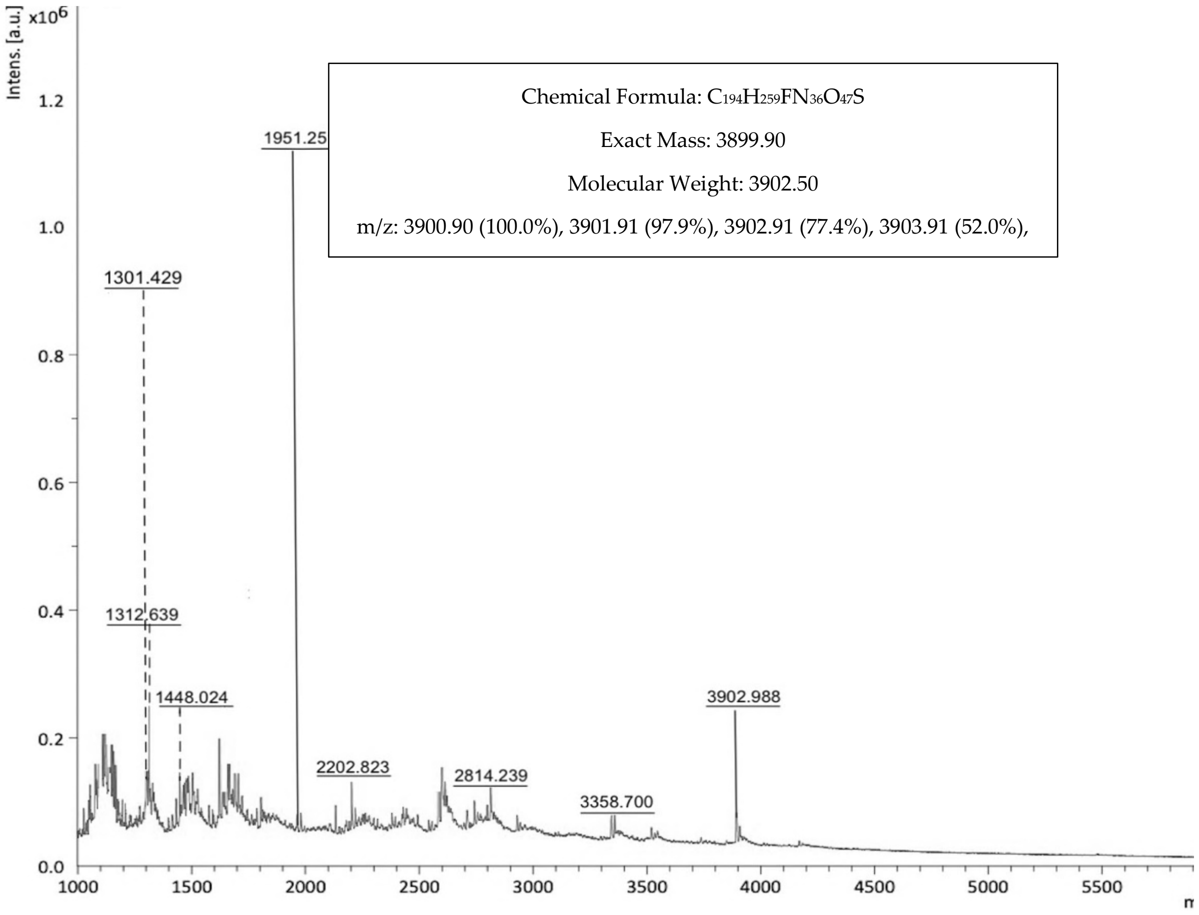

Figure S14. MALDI-TOF mass spectrum of T7-SN-38-Rucaparib conjugate (**12**) m/z = 3902.98; mass/2 = 1951.25; mass/3 = 1301.42.

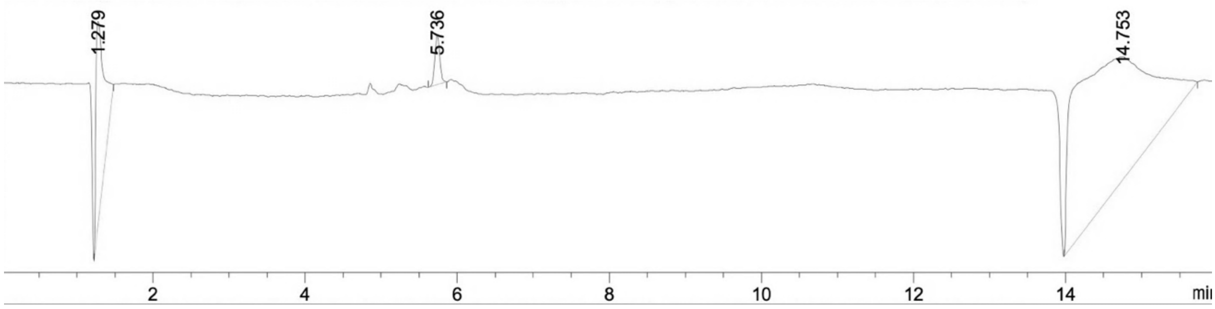

Figure S15. RP-HPLC analysis of T7-SN-38-Rucaparib (**12**) showing isolation of a pure compound with retention time 5.73 min.
